# The evolution of tit-for-tat in bacteria via the type VI secretion system

**DOI:** 10.1038/s41467-020-19017-z

**Published:** 2020-10-26

**Authors:** William P. J. Smith, Maj Brodmann, Daniel Unterweger, Yohan Davit, Laurie E. Comstock, Marek Basler, Kevin R. Foster

**Affiliations:** 1 Department of Zoology, University of Oxford, Oxford, OX1 3SZ UK; 2 Department of Biochemistry, University of Oxford, Oxford, OX1 3QU UK; 3 Biozentrum, University of Basel, Klingelbergstrasse 50/70, CH-4056 Basel, Switzerland; 4 Institute for Experimental Medicine, Kiel University, 24105 Kiel, Germany; 5 Max-Planck Institute for Evolutionary Biology, 24306 Plön, Germany; 6 Institut de Mécanique des Fluides de Toulouse, CNRS and Université de Toulouse, 31400 Toulouse, France; 7 Division of Infectious Diseases, Brigham and Women’s Hospital, Boston, MA 02115 USA

**Keywords:** Computational models, Evolutionary theory, Bacterial evolution, Bacterial secretion, Bacterial toxins

## Abstract

Tit-for-tat is a familiar principle from animal behavior: individuals respond in kind to being helped or harmed by others. Remarkably some bacteria appear to display tit-for-tat behavior, but how this evolved is not understood. Here we combine evolutionary game theory with agent-based modelling of bacterial tit-for-tat, whereby cells stab rivals with poisoned needles (the type VI secretion system) after being stabbed themselves. Our modelling shows tit-for-tat retaliation is a surprisingly poor evolutionary strategy, because tit-for-tat cells lack the first-strike advantage of preemptive attackers. However, if cells retaliate strongly and fire back multiple times, we find that reciprocation is highly effective. We test our predictions by competing *Pseudomonas aeruginosa* (a tit-for-tat species) with *Vibrio cholerae* (random-firing), revealing that *P. aeruginosa* does indeed fire multiple times per incoming attack. Our work suggests bacterial competition has led to a particular form of reciprocation, where the principle is that of strong retaliation, or ‘tits-for-tat’.

## Abstract (web summary)

Game theory has contributed much to the understanding of social evolution. In an elegant combination of experimental tests and modelling, this study suggests that when bacteria face intense competition, repeated retaliation outcompetes a single tit-for-tat response to attack.

## Introduction

The type VI secretion system (T6SS) is a contact-dependent bacterial weapon that is found in numerous bacterial species^1–4^ and used to inject toxic effector proteins into neighboring cells^5–7^. Structurally and functionally homologous to a phage’s tail^8^, the T6SS consists of a membrane-bound baseplate complex, an effector-tipped needle, and a surrounding sheath whose contraction drives the needle through the membranes of target cells^9,10^. Used by many notorious plant and animal pathogens, the T6SS is a potent anti-competitor weapon: the T6SS can determine whether a strain can invade, or defend, its niche in both environmental and host-associated microbial communities^11–16^.

There is remarkable variation in the regulation and use of T6SS weaponry across species. Bacteria activate and deploy the T6SS across a range of environmental contexts^16–19^ and against both prokaryotic and eukaryotic targets^20–22^. The specific pattern of firing by cells also varies: whereas placement of T6SS assembly appears to be random in some species, such as *Vibrio cholerae*, *Serratia marcescens*, and *Acinetobacter baylyi*^23–25^, other bacteria are known to fire from specific locations on their cell membranes. Perhaps, the most striking example of this spatiotemporal control is the retaliatory firing strategy observed in *Pseudomonas aeruginosa*, whose T6SS apparatus (encoded at the HSI-1 locus) is specifically activated in cells that are themselves attacked by T6SS needles^26,27^.

The regulatory pathway underpinning T6SS retaliation in *P. aeruginosa* is an active topic of study. So far, it has been shown that various stimuli can trigger counterattacks in *P. aeruginosa* strain PAO1, including incoming T6SS attacks from multiple bacterial species^23,27,28^, conjugative T4SS pili^29^, and membrane-disrupting antibiotics like polymyxin B^29^. There is also evidence that retaliation can be toxin-specific—not all T6SS effectors trigger counterattacks, and in *V. cholerae*, only the lipase effector TseL triggers retaliation^30^. Across stimuli, *P. aeruginosa* appears to be responding to membrane perturbation, and a putative model is that this response is mediated post-transcriptionally via the TagQRST pathway. This signaling cascade leads to the localized phosphorylation of cytoplasmic Fha1 proteins, and subsequent T6SS activation^31–33^.

Although the molecular regulation of retaliatory T6SS firing has received attention^27,28,30,34^, its evolution has not—leaving open the question of why such a complex strategy has evolved in bacteria, and only in some species. At a broader level, while the evolution of reciprocation has a long history of study in evolutionary biology^35–38^, past efforts have focused largely on the evolution of reciprocal cooperation, rather than competition. Understanding the evolution of T6SS regulation and retaliation is therefore important, both for understanding bacterial warfare and as a distinct case in evolutionary biology.

To address this, we used an agent-based modeling framework to simulate competition between different T6SS strategists. By combining modeling with game theory, we explore the evolution of T6SS regulation, including tit-for-tat (TFT) firing, across a wide range of conditions. This reveals that TFT has significant limitations as a strategy for T6SS warfare. We found that it rarely wins in direct competition because it fails to fire against unarmed strains and always fires second against armed strains. However, we also found that a strong retaliator, which fires multiple times in response to an attack, is a powerful competitor against randomly firing T6SS attackers. Finally, by studying the retaliatory firing patterns of *P. aeruginosa*, we show that it does indeed fire multiple times in response to an incoming attack. Our work suggests that T6SS reciprocation is most beneficial during combat when performed in an aggressive manner.

## Results

### Agent-based modeling of different T6SS firing strategies

To study the interactions and evolution of different T6SS firing strategies, we began with an established agent-based modeling framework (CellModeller)^39–42^. The heart of this model is a realistic representation of physically interacting bacteria growing in dense communities. Agent-based models of this sort have proved to be a powerful means to explore cell–cell interactions in bacterial communities, generating a wide range of predictions that have been verified by empirical work (reviewed in ref. ^43^).

We recently reported a new version of this model^44^, designed specifically for the study of T6SS competition, in which cells can intoxicate neighbors by firing T6SS needles. Here, we extend this model, such that different modes of T6SS firing (Supplementary Table 1) can now be represented and compared: cells can be programmed not to fire, or to fire constantly and in random directions, or to fire in more elaborate patterns. Using this tool, one can then compare the effectiveness of T6SS firing strategies observed across different bacterial species, under tightly controlled conditions, while varying physiological parameters (T6SS firing rate *k*_fire_, carriage cost *c*_upfront_, pro rata cost *c*, and hit resilience *N*_hits_). Further details of our model are provided in the “Methods” section.

### Random T6SS firing is effective against unarmed strains

First, we used our agent-based model to study competition between bacterial strains with two basic strategies: Random-firing T6SS+ attackers (R) and T6SS-susceptible Unarmed cells (U). We simulated community growth within 2-D patch environments, beginning with a randomly scattered, 1:1 mixture of R and U cells. Each patch simulation begins with a finite, uniform resource quota that is consumed as cells grow (exponentially, at rate *k*_grow_), and simulations end once a patch becomes depleted of resources (Supplementary Fig. 1A). Would-be weapon users therefore face a trade-off: attacking one’s competitors prevents them from using up a patch’s resources, but at the costs of both reduced reproductive rate and efficiency. Here and throughout, we assume that T6SS+ strategists are immune to the toxins of their clonemates. We also assume that possessing and expressing T6SS genes is costly, such that the specific growth rate of a T6SS+ strain is reduced in proportion to its firing rate. However, we later show that our key conclusions do not rest upon the assumption that the T6SS is costly.

**Fig. 1:**
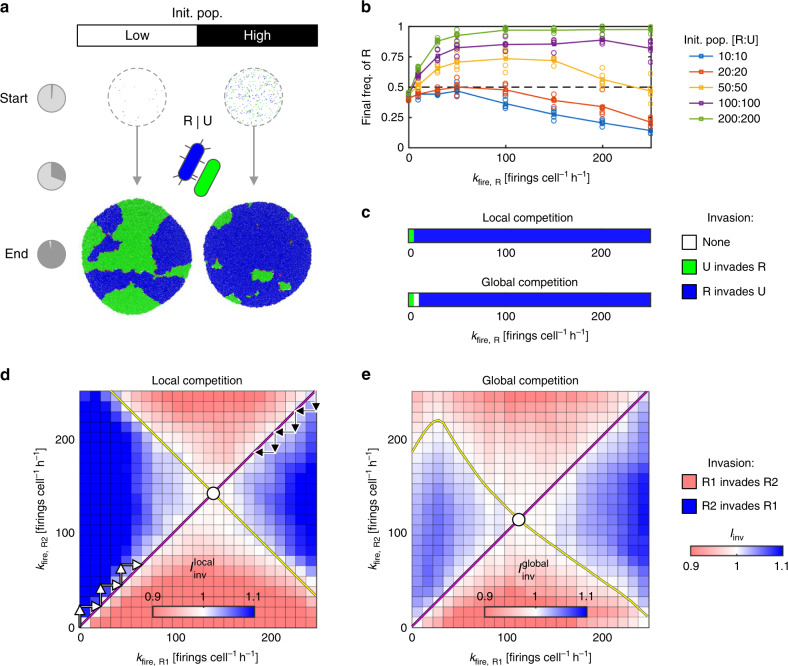
The evolution of random T6SS firing. **a** Simulation snapshots showing initial and final cell configurations for surficial competition between T6SS− Unarmed strain (U, green) and a Random-firing T6SS+ strain (R, blue). Simulations are carried out for both low and high initial cell densities (left and right columns; initial cell populations 10 vs. 10 and 200 vs. 200 cells, respectively); pie charts (left) indicate the consumption of patch resources. Firing rate *k*_fire,R_ = 50 firings cell^−1^ h^−1^. **b** Competition outcomes, measured by final R cell proportion, as a function of firing rate *k*_fire,R_ for increasing initial cell densities (see legend, right). Circles and lines correspond to data points and their means, respectively. **c** Invasion plots showing outcomes of local and global invasion analyses for R vs. U competition (see “Methods”), as a function of firing rate *k*_fire,R_, for high initial cell density (200 vs. 200 cells); additional cases are shown in Supplementary Fig. 3. **d**, **e** Pairwise invasion plots for competing R-type strategists (R1, R2), showing invasion outcomes for local (**d**) and global (**e**) competition scales for intermediate cell density (50 vs. 50 cells). Arrows illustrate progression of evolving firing rates *k*_fire,R_, converging on evolutionary stable strategy firing rates (ESS, white circles). Simulation parameters used throughout: *N*_hits_ = 2, *c* = 0.001; *n* = 5 simulation replicates per case in **b**, **c** and *n* = 10 per case in **d**, **e**. Source data are provided as a Source Data file.

Figure 1a shows two patch simulations in which bacterial strains with R and U strategies compete, carried out for different starting cell densities. In the left example (at low cell density), T6SS-mediated killing marginally increases the final frequency of R strategists; to the right (high cell density), this competitive advantage is greatly enhanced. Strong density dependence is consistent with previous studies of T6SS competition—higher cell density results in increased (and earlier) contact between R and U cells, increasing overall killing^45^. Another benchmark of the model is that we also observe T6SS activity resulting in increased spatial segregation between competing strains (Fig. 1a and Supplementary Movie 2), compared with T6SS− controls (Supplementary Fig. 1A and Supplementary Movie 1), which is consistent with previous theoretical and empirical work^46^.

To further explore the competitive value of Random T6SS firing, we compared R vs. U competition outcomes for a wide range of input parameters: varying initial cell density, T6SS firing rate, weapon cost, lysis rate, and toxin potency. These analyses confirmed that Random T6SS firing can indeed offer a competitive advantage (evidenced by increased R frequency after competition) under a broad set of conditions. As well as being favored by high cell density (Fig. 1b, Supplementary Figs. 2 and 7, and Supplementary Movie 2), which produces greater contact between R and U cells, natural selection for Random T6SS attackers is increased for low weapon costs (Supplementary Figs. 1C, D and 7), high toxin potency (Supplementary Figs. 1E and 2), and low victim cell lysis duration (Supplementary Fig. 1E). Similarly, we found that reducing weapon costs generally increases the optimal T6SS firing rate (Supplementary Fig. 7).

Our models confirm the intuition that the T6SS can help a bacterial strain to increase its frequency within a patch. But are Random T6SS attackers also expected to invade an Unarmed population when one also considers the competition between different patches to colonize new sites (global competition)? This question is important because while aggression may allow a strain to defeat a competitor, if this comes at a large personal cost, an aggressive strain may still end up producing very few dispersing cells. If other patches contain only passive strategies that make many dispersing cells, therefore, an aggressive strategy could win locally but lose globally by failing to colonize new patches. To address this question, we embedded our model in a game-theoretic framework that uses the principles of adaptive dynamics^47^. As detailed in the “Methods” section, this approach considers the fate of an initially rare, novel strategist placed in a metapopulation (large set of patches) dominated by another, resident strategist. If the relative fitness of the novel strategist is greater than that of the resident, we assume that its frequency in the metapopulation will increase until it supplants the resident as the common strategy. We then also check whether the resident can itself re-invade a population of the novel strategist from rarity, and when it cannot, we assume that the novel strategy will permanently replace the resident.

Figure 1c shows the fate of an invading Random T6SS attacker (summarized by an invasion index, *I*_inv_) as a function of its attack rate, *k*_fire,R_, for when there is purely local competition (“local competition”) and when there is both local and global competition (“global competition”, see “Methods”). For local competition, Random attackers compete only within patches with the resident Unarmed strain. For global competition, they must also compete with Unarmed cells in neighboring patches where Random attackers are absent. In both scenarios, we find that R can successfully invade U for all non-zero firing rates, assuming a high initial cell density (Fig. 1c, 200:200 cells). For lower cell densities, the range of viable *k*_fire,R_ values narrows, and is generally smaller for global competition than for local competition (Supplementary Fig. 3). In sum, in this system, we find that local and global competition scenarios give qualitatively similar results.

### An evolutionarily stable rate of random T6SS firing

Our models predict that Random T6SS attackers will readily invade a population of Unarmed cells, under a range of conditions. As Random attackers become more abundant, they will begin to encounter one another, and so we next consider the outcomes of battles between different R-type strategists. When can one Random attacker invade another’s patch? Here we studied competition between pairs of R-type strategists, R1 and R2, each having its own attack rate *k*_fire,R1_, *k*_fire,R2_, and each being susceptible to the other’s toxins. Figure 1d shows a pairwise invasion plot, commonly used in adaptive dynamics^47^, indicating which of R1 and R2 invades the other as a function of their respective attack rates, for local (within-patch) competition. We find that either competitor can invade the other by firing faster than it (Supplementary Movie 3 shows an example of this), but only up to a point. Beyond the yellow diagonal line, having a higher attack rate than one’s competitor makes one vulnerable to invasion, since the increased costs of the higher attack rate outweigh any additional benefits conferred^44^. Figure 1e shows a similar pairwise invasion plot, this time computed for the case of global competition.

What firing rate *k*_fire,R_ is predicted to evolve during competition between Random T6SS attackers? Here, we compute the evolutionarily stable strategy (ESS) as the value of *k*_fire,R_, denoted $k_{{\mathrm{fire}},{\mathrm{R}}}^{{\mathrm{ESS}}}$, for which a resident strategist cannot be invaded by mutants with a higher or lower firing rate. In Fig. 1d, the ESS for local R1 vs. R2 competition is shown as a white circle—if a resident strategist (R1) adopts this firing rate, then a mutant strategist (R2) cannot invade irrespective of its firing rate.

Moreover, one can show that any resident population will evolve towards this strategy. For example, suppose we begin with a resident R1, which possesses the T6SS but does not use it (*k*_fire,R1_ = 0, Fig. 1d). Then suppose a mutant R2 appears in this population with *k*_fire,R2_ = *δ*, where *δ* > 0 represents some small increment in firing rate. Since the local invasion index $I_{{\mathrm{inv}}}^{{\mathrm{local}}}\left( {0,\,\delta } \right)\, > \, 1,$ R2 can invade R1, and *k*_fire,R1_ = *δ* becomes the resident strategy. The same outcome occurs with *k*_fire,R2_ = 2*δ*, 3*δ* … such that successive invasions by incrementally more aggressive mutants increase the firing rate in the resident population (Fig. 1d, black arrows), eventually converging on $k_{{\mathrm{fire}},{\mathrm{R}}}^{{\mathrm{ESS}},{\mathrm{local}}}$. Similarly, a resident population with a very high firing rate (e.g., *k*_fire,R1_ = 250 firings cell^−1^ h^−1^) will be displaced by mutants with lower firing rates (Fig. 1d, yellow arrows), again converging on $k_{{\mathrm{fire}},{\mathrm{R}}}^{{\mathrm{ESS}},{\mathrm{local}}}$. Global competition (Fig. 1e) favors a reduced level of aggression than local competition (i.e., $k_{{\mathrm{fire}},{\mathrm{R}}}^{{\mathrm{ESS}},{\mathrm{local}}}$ > $k_{{\mathrm{fire}},{\mathrm{R}}}^{{\mathrm{ESS}},{\mathrm{global}}}$), a trend also seen for other strategist pairs at various initial densities (Supplementary Fig. 3). This follows one of the core results of social evolution: between-group selection can select against competition, and for cooperation, because global (between-group) competition makes group productivity important for fitness^48,49^.

### TFT retaliation fails to beat a random attacker

Our results indicate that Random T6SS firing can often be a successful strategy, both enabling invasion of Unarmed populations and achieving higher cell frequencies against other Random T6SS attackers than Unarmed strategists attain. From this baseline, we can evaluate the evolutionary costs and benefits of the more complex T6SS firing strategy of TFT. Based on published empirical work on *P. aeruginosa*^23,27^, we assume that TFT differs from R in two key respects: (i) TFT does not fire its T6SS continuously, but counterattacks once per incoming attack (retaliatory firing); (ii) TFT does not fire from randomly chosen sites on its cell membrane, but instead from the points where incoming attacks struck (spatial sensing). To provide a fair basis for strategy comparison, we assume TFT to be identical to R in all other respects (toxin potency, lysis delay, weapon costs per T6SS firing, costs of weapon carriage).

**Fig. 2:**
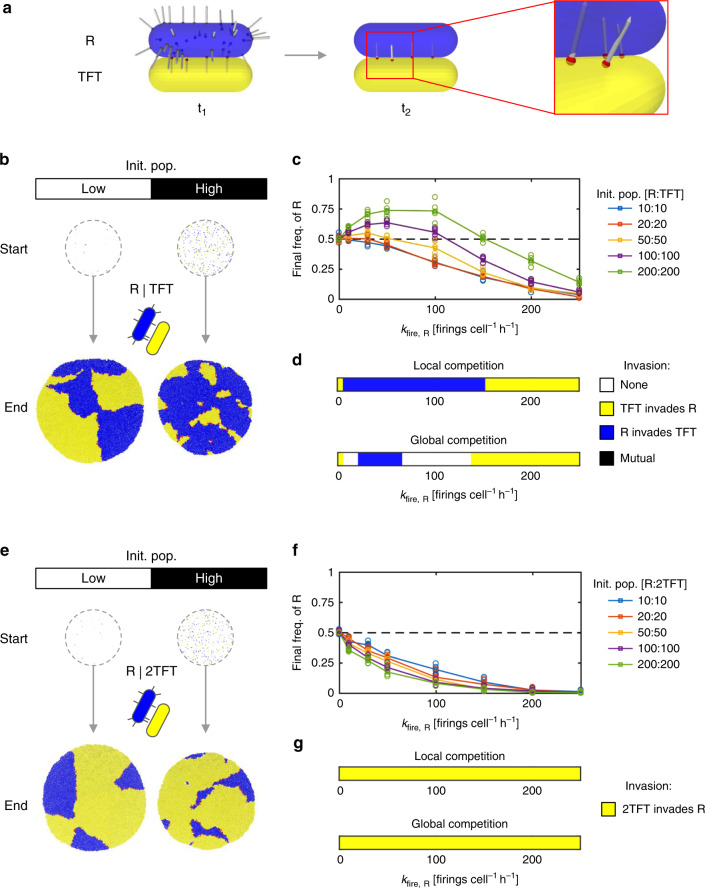
2-Tits-for-tat (2TFT) outperforms tit-for-tat (TFT) vs. a (R)andom firing strategy. **a** Model representation of retaliatory T6SS firing in response to a random attacker (R, blue). Following R’s initial attack (*t*_1_), the retaliator cell (TFT, yellow) fires T6SS needles outwards from the points on its surface where initial attacks struck (*t*_2_, magnified box). **b** Simulation snapshots showing initial and final cell configurations for competitions between R and TFT strategists (“low” and “high” initial cell populations correspond to 10 vs. 10 and 200 vs. 200 cells as in Fig. 1). **c** Competition outcome, measured by final R cell proportion, as a function of firing rate *k*_fire,R_ for increasing initial cell densities (see legend, right). Circles and lines correspond to data points and their means, respectively. **d** Invasion plots showing outcomes of local and global invasion analyses for R vs. TFT competition, as a function of firing rate, *k*_fire,R_, for high initial cell density (200 vs. 200 cells). **e**–**g** Analogous to **b**–**d**, except with TFT replaced by 2TFT, which counterattacks twice per successful oncoming attack. Simulation parameters: *N*_hits_ = 2, *c* = 0.001. *n* = 5 simulation replicates per case in **c**, **d**, **f**, and **g**. Source data are provided as a Source Data file.

Figure 2a shows our implementation of a TFT strategist in the agent-based model. To assess conditions favoring TFT strategists, we competed TFT against R for different initial cell densities, as before (Fig. 2b, c, Supplementary Fig. 2, and Supplementary Movie 4). We were surprised to find that, while TFT generally does better against R than U (cf. Figure 1b), R is nevertheless predicted to outcompete TFT in a wide range of conditions. Specifically, we see that R can always evolve to a firing rate *k*_fire,R_ that makes it equal or better than TFT (Fig. 2d). We also see that it is at the higher initial cell densities that R performs the best against TFT. This effect is telling: increasing initial cell density simultaneously creates more fronts between competing cell groups *and* increases the time for which competing strains are in physical contact—both of which favor the strain with the best contact-dependent attack (above, Fig. 1a). Overall, our model suggests that R can invade and displace TFT simply by evolving relatively low *k*_fire,R_ values, for both local and global competition scales (Fig. 2d and Supplementary Figs. 2 and 3), provided cell density is sufficient.

### Retaliation can evolve by investing in attack and defense

We found that a wide range of conditions preclude the evolution of TFT retaliatory T6SS firing from a population of Random attackers. Trivially, TFT is also guaranteed to lose against U, since the latter never triggers retaliatory T6SS attacks, and is spared the cost of T6SS carriage^24^. How then could TFT have evolved in *P. aeruginosa* if it is predicted to be typically outcompeted by other less sophisticated strategies?

To resolve this apparent paradox, we considered ways in which the TFT strategist might evolve to improve its competitive ability. This revealed that increasing the number of counterattacks launched by retaliators can pay great dividends. Specifically, we found that a strong retaliator strategist—dubbed 2-tits-for-tat (2TFT)—is highly successful against a Random T6SS attacker (Fig. 2e), outcompeting it for all T6SS firing rates and cell densities studied (Fig. 2f and Supplementary Movie 5). Swapping TFT for 2TFT also reversed the trend in competition outcome with respect to initial cell density, with higher cell densities now favoring 2TFT instead of R (Fig. 2f, cf. Fig. 2c). This again illustrates that high cell density tends to intensify contact-dependent warfare, and thereby favor whichever strain has the best contact-dependent attack (Fig. 1a, last section).

Accordingly, we also found that 2TFT is able to invade a population of R cells for all *k*_fire,R_ > 0 (Fig. 2g), and for all cell densities studied (Supplementary Fig. 3). However, this robust competitive advantage disappeared when we reduced the resilience of both strategists (*N*_hits_ reduced to 1 from 2), such that a single T6SS hit is sufficient to kill any non-clonemate cell (Supplementary Fig. 3): here, 2TFT performs no better than TFT. This suggests that sufficient investment in both defense against T6SS intoxication and attack via increased firing is important for the evolution of retaliation.

While 2TFT is very successful in competition with R cells, it is expected to lose against unarmed (U) strains, just like TFT. This raises the possibility of rock-paper-scissors dynamics, also suggested in a recent study^50^, where non-transitive interactions between competing bacterial species stabilizes variation in T6SS firing patterns^51,52^. Consistent with this possibility, we found that parameter combinations exist (Supplementary Fig. 5) where unarmed U strains are beaten by random R attackers (T6SS killing trumps growth advantage), who are beaten by 2TFT (superior killing and growth advantage trumps T6SS aggression), and who can be beaten in turn by unarmed strains (growth advantage trumps unused costly T6SS).

We also tested the robustness of 2TFT’s supremacy across a range of additional biological scenarios, including low diversity in T6SS toxins in the population (Supplementary Fig. 8), the potential for cheating strategies that do not use the T6SS but which are immune to some T6SS toxins (Supplementary Figs. 9 and 10), and conditions with high within-patch relatedness (Supplementary Figs. 11 and 12). We discuss the effects of these scenarios in detail in the supplement, but across all conditions, 2TFT was predicted to be equivalent or superior to both TFT and R.

### Retaliation brings both geometric and economic benefits

**Fig. 3:**
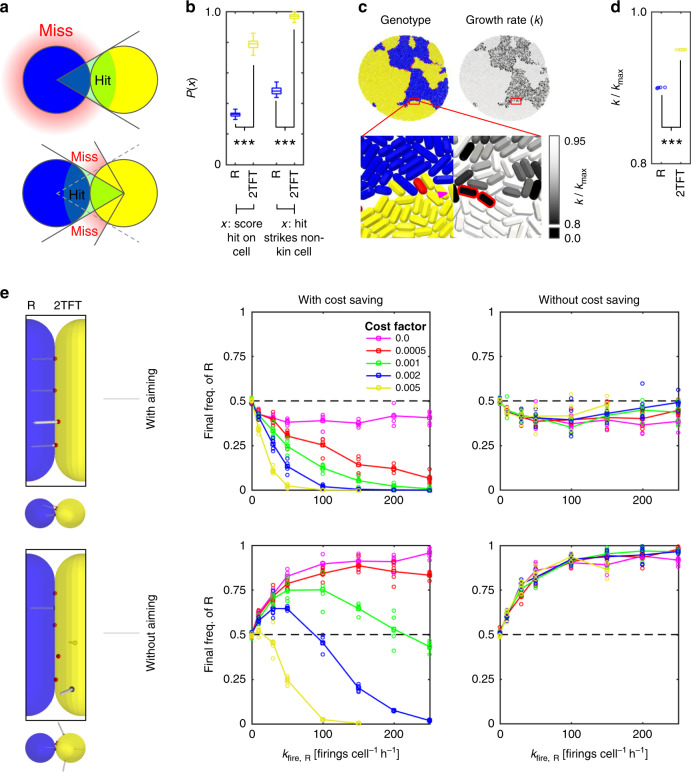
Aiming and cost saving allow 2TFT to beat random attackers. **a** Diagram comparing likelihood of successful T6SS attack for random firing (top) and retaliatory firing (bottom). **b** Measurements of absolute and non-clonemate cell hit probabilities from static, mixed-cell populations, for random (R, blue) and retaliatory (2TFT, yellow) T6SS firing. Statistical test for absolute hit probability comparison: two-sample, two-sided *t* test, no adjustment for multiple comparisons, *t* = −125.0560, effect size (Cohen’s *d*) = 17.676539, *p* = 2.2803e − 190. For comparison of non-clonemate cell hit probabilities, we use the same test; here *t* = −160.6783, effect size 22.6885, *p* = 1.0388e − 211. In both cases, *n* = 100 firing events taken from same sample, giving 198 degrees of freedom. Whiskers, boxes, and centerlines denote ranges, interquartile ranges, and median values, respectively. **c** Visual comparison of R and 2TFT cell growth rates during competition. Cell configuration and magnified sections are colored by cell type (left) or by growth rate (right). Magenta arrow highlights a single TFT cell whose growth rate is reduced by active firing; dead cells are outlined in red in the right-hand panel. **d** Comparison of R and 2TFT cell population average growth rates, measured at the end of five separate R vs. 2TFT competitions (statistical test as in **b**; *t* = −207.4396, effect size 131.3978, *p* = 3.2643e − 16, growth rate from ~10,000 cells across five independent simulations, 8 degrees of freedom). Circle markers indicate population means. **e** Comparison of R vs. 2TFT competition outcomes, in which 2TFT strategists are modified to remove T6SS aiming (bottom row) and/or cost saving (right column), for increasing weapon costs *c* (see legend, top left). Circles and lines correspond to data points and their means, respectively. In **b**, **d**, ***p < 0.001. Source data are provided as a Source Data file.

We next sought to characterize the origin of 2TFT’s advantage over R, in contrast to the standard model of T6SS retaliation (TFT). We identified two key advantages offered by retaliatory T6SS firing, and used our models to compare their relative contributions to 2TFT’s fitness in competition with R (Fig. 3). First, the ability to sense *where* incoming attacks are coming from allows T6SS counterattacks to be aimed specifically at attackers. By contrast, Random attackers have no information on where target cells are^9^, and so miss most of the time (Fig. 3a). We confirmed this principle by measuring T6SS hit:miss ratios in fixed, well-mixed configurations of cells, showing that attacks by 2TFT cells were significantly more likely to hit R cells than vice versa (Fig. 3b, see “Methods”).

Second, the ability to sense *when* one is being attacked prevents costly use of the T6SS when it is not needed. Examination of cell growth rates during R vs. 2TFT competitions confirmed that only 2TFT cells that are in contact with competitors pay for T6SS firing—compared with R cells, which pay for constant T6SS firing whether or not competitors are actually in range (Fig. 3c). We found that this resulted in significantly higher specific growth rates for 2TFT cells than for R cells (Fig. 3d).

To determine which of these advantages—improved aim or lower cost—drives 2TFT’s success in a given scenario, we created three new retaliator phenotypes with one or both advantages removed (Fig. 3e). To remove the advantage of T6SS aiming through spatial sensing, we configured 2TFT cells to counterattack from randomly chosen sites on their membranes, instead of from the points at which incoming attacks struck (Fig. 3e, bottom row). To remove the advantage of reduced T6SS cost, we configured 2TFT cells to pay the same growth costs as Random T6SS attackers, for any given attack rate *k*_fire,R_ (Fig. 3e, right column). Comparing the single knockout cases (loss of aiming or loss of cost saving) against a normal R vs. 2TFT competition, we found that removing cost saving still allowed 2TFT to beat R (albeit by a reduced margin) irrespective of weapon cost factor *c*, including the limit in which weapon use is cost-free (Fig. 3e, top right). By contrast, eliminating only T6SS aiming (Fig. 3e, bottom left) allowed R to beat 2TFT, except where weapon costs were very high. Similar results were seen when cell density was varied instead of weapon costs (Supplementary Fig. 4). In sum, a 2TFT strategist can accrue benefits from both advantages, but it is improved aim that appears most critical to their success.

### *Pseudomonas aeruginosa* launches multiple counterattacks

Our model suggests that the evolution of retaliation via the T6SS rests upon at least three specific characteristics of a retaliating cell: (1) intrinsic resistance to T6SS attack such that a cell can survive more than one hit (Supplementary Fig. 3), (2) the ability to reciprocate with multiple counterattacks (Fig. 2), and (3) the ability to aim counterattacks towards aggressors (Fig. 3). Predictions 1 and 3 are already supported by published work. An opportunistic pathogen found in a wide variety of environments, *P. aeruginosa* is a notably resilient species with a high natural tolerance to many antibiotics and other toxins^53^. More specifically, *P. aeruginosa* cells are known to regularly tolerate multiple hits from the T6SSs of other species, including the human pathogen *V. cholerae*^27^. In addition, the firing behavior of *P. aeruginosa* in response to an incoming hit is visibly non-random, occurring reliably on the same side of the cell as the incoming hit^23^. However, prediction 2 has not been examined empirically, offering us an opportunity to test our model against an unknown aspect of T6SS biology.

We therefore analyzed the T6SS counterattacks of *P. aeruginosa* (strain PAO1) cells, in response to random attacks by *V. cholerae* (strain 2740-80) bacteria, as in the original T6SS retaliation study^27^ and subsequent work^29,54–56^. In our experiments, both cell types express functional T6SS apparatus, the sheaths of which (TssB subunits in the case of *P. aeruginosa* and VipA subunits in the case of *V. cholerae*) carry fluorescent tags (see “Methods”). These tags allow individual T6SS firing events to be tracked using time-lapse fluorescence microscopy, as described in previous studies^25,27,57^. When the two are grown together on agarose pads, *V. cholerae* antagonizes *P. aeruginosa* and causes it to launch counterattacks (Supplementary Fig. 6 and Supplementary Movie 6), such that T6SS dynamics of the two species can be compared directly in the same setting. By contrast, control experiments using a T6SS− *V. cholerae* mutant resulted in no *P. aeruginosa* T6SS activity, reproducing behavior reported in previous studies^27–29^, and confirming the retaliatory nature of *P. aeruginosa* attacks (Supplementary Movie 7).

**Fig. 4:**
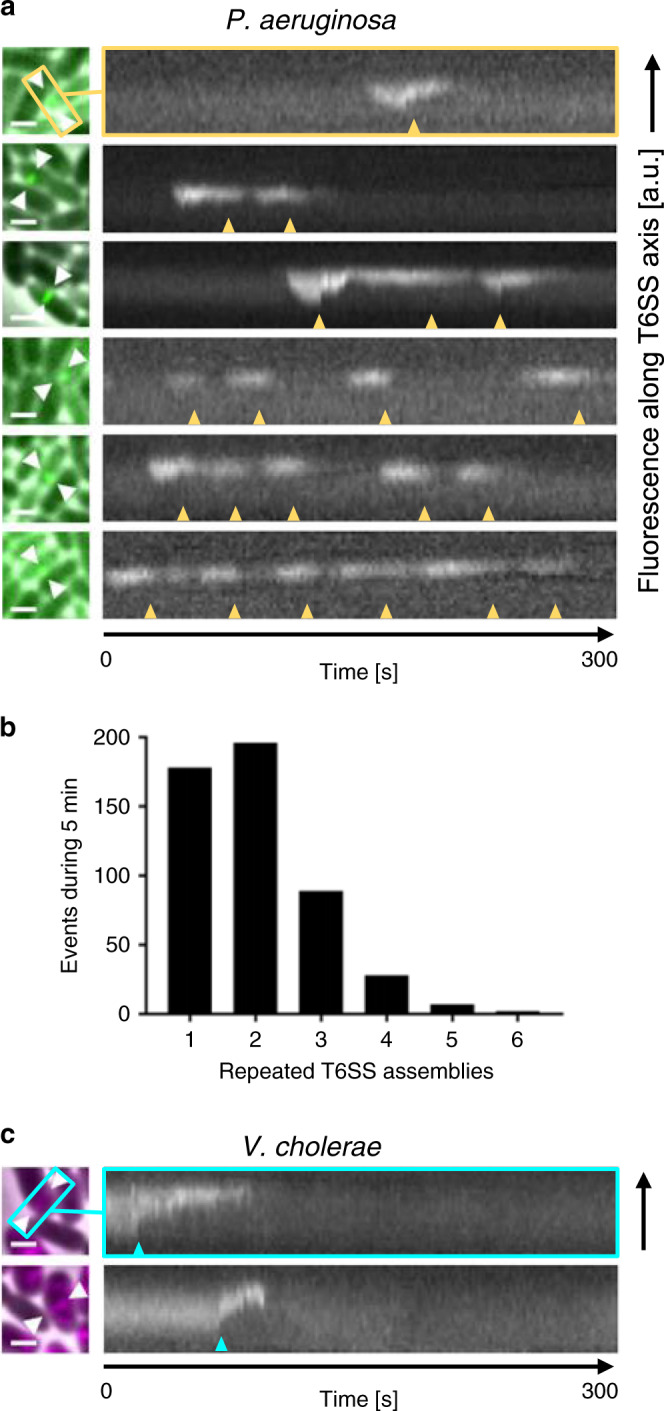
Repeated T6SS assemblies in *P. aeruginosa*. **a** Mixture of *P. aeruginosa* PAO1 *tssB-mNeonGreen* (green) with T6SS+ *V. cholerae* 2740‐80 *vipA-mCherry2* (black), highlighting retaliatory T6SS contractions within *P. aeruginosa* cells. A merge of phase contrast and GFP channels is shown (left). Field of view, 3.3 × 3.3 µm^2^, is shown; scale bars, 1 µm. White arrows mark axes of assembled T6SSs. Kymograms (right) show fluorescence signal along each T6SS axis (2 s pixel^−1^, GFP channel), indicating up to six T6SS contractions occurring at the same location over a 5-min period (yellow arrows). Vertical axes correspond to direction of T6SS contraction. **b** Histogram showing numbers of repeated T6SS contractions of *P. aeruginosa* PAO1 *tssB-mNeonGreen* cells in contact with *V. cholerae* 2740‐80 *vipA-mCherry2* (average of repeated firings = 1.992, standard deviation of 0.975, median: 2, *n* = 500, two biological replicates). **c** Kymograms recorded for *V. cholerae* cells, analogous to **a**, showed no repeated T6SS contractions within the 5 min viewing period (cyan arrows). Scale bars, 1 µm. Source data are provided as a Source Data file.

Figure 4 shows example kymographs, tracking sheath lengths in individual T6SS apparatus imaged in *P. aeruginosa* cells (Fig. 4a) during these co-culture experiments. We observed that, following an incoming attack, *P. aeruginosa* cells fire repeatedly (between 1 and 6 firings over a 5-min time-lapse, with median 2 firings per site; see Fig. 4b and Supplementary Movie 8). As predicted by our model, therefore, we found that retaliatory firing in *P. aeruginosa* is associated with multiple counterattacks from the same T6SS site. Conversely, we could detect no instances of repeated T6SS firing by *V. cholerae* within the same time window (Fig. 4c), confirming that repeated T6SS firing is not simply a universal trait among γ-Proteobacteria.

## Discussion

We found that random constitutive firing of the T6SS can readily evolve in unarmed populations, provided that (i) weapon costs are not excessive and (ii) initial mixing provides enough inter-strain contact. By contrast, retaliatory firing is successful only against other T6SS users, and then only if the retaliator is robust to incoming attack, gains an aiming advantage, and deals more damage than it sustains (strong retaliation). Ultimately, these additional constraints stem from the first-strike advantage possessed by random attackers: having already been struck by at least one T6SS needle, a retaliator always enters combat at a disadvantage, requiring that retaliation be disproportionate to be generally successful.

The additional constraints limiting retaliator evolution may explain why *P. aeruginosa* is, to our knowledge, the only example of a T6SS retaliator found so far—whereas many species appear to use random T6SS firing^23–25^. It is also clear that *P. aeruginosa* is very well suited for T6SS retaliation. First, *P. aeruginosa* can resist oncoming T6SS attacks from other species like *V. cholerae*^27^. Second, *P. aeruginosa*’s ability to aim T6SS firing—through spatially resolved, TagQRST-mediated attack sensing—is likely to be a key contributor to its success as a retalitor (Fig. 3e), because it provides cells with additional information on the location of attackers. This contrasts with other forms of T6SS regulation, where the rate of T6SS firing is increased in response to cellular damage^18,24,58^, but with T6SS placement occurring apparently at random. By placing T6SS assemblies at attack sites, *P. aeruginosa* can substantially improve its hit efficiency, compared with a random firer that has no information on the location of its target.

Third, our models predict that *P. aeruginosa* cells can only fully exploit this aiming advantage if they also launch multiple counterattacks from a given site of impact. Otherwise, they still stand to lose more cells per pairwise T6SS battle than their competitors, such that the latter can still win overall if the two strains are sufficiently well mixed. Our experiments confirmed that *P. aeruginosa* does indeed fire repeatedly from T6SS assemblies placed at hit sites, a pattern not observed in random-firing *V. cholerae*. In light of this observation, *P. aeruginosa’s* retaliatory firing appears better characterised as a *tits*-for-tat strategy than a *tit*-for-tat one^36^.

## Methods

### Agent-based model

As in previous studies^39–41^, we model bacterial communities as collections of 3-D rod-shaped cells, growing in independent patch environments on a flat surface (Supplementary Fig. 1A). Every cell is an independent agent whose behavior depends on its phenotype, and on its interactions with neighboring cells. Each model simulation tracks cell growth, movement, and death within a single patch. Patches have an allotted quota (*E*_0_) of growth-limiting resources, which cells consume until the patch becomes depleted, thereby ending the simulation. Cell phenotypes, model variables, and model parameters are summarized in Supplementary Tables 1–3, respectively.

*Cell growth and division*: Each cell’s volume *V*_*i*_ increases exponentially through elongation, from initial volume *V*_0_, according to the equation d*V*_*i*_/d*t* = *k*_grow,*i*_*V*_*i*_, where *k*_grow,*i*_ is a (phenotype-dependent) cell growth rate with maximum *k*_max_. For simplicity, we assume that all living cells deplete patch resources *E* at a rate proportional to their current volume, and independent of the resources remaining in the patch (i.e., zeroth-order kinetics): d*E*/d*t*  = −*k*_max,_∑_*i*_*V*_*i*_. Cells divide lengthwise into two identical daughter cells once they reach volume 2*V*_0_ + *η*_division_, with *η*_division_ a uniform random noise term. Each daughter’s axis vector ${\hat{\boldsymbol{a}}}_i$ is perturbed slightly by a noise term with weight *η*_orientations_, to represent spatial imperfections in the division process. Following the cell growth phase, the cell configuration is returned to a quasi-stationary mechanical equilibrium using an energy minimization algorithm, described previously^39^. Briefly, cells whose surfaces overlap are identified using a standard contact finding algorithm^59^. Then, sets of impulse vectors ***p*** satisfying the equation $\left( {A^{\mathrm{T}}A + \alpha M} \right){\boldsymbol{p}} = - A{\boldsymbol{d}}$ are calculated, where *A* is a matrix summarizing cell contact geometry, *M* is a matrix of drag coefficients, ***d*** is a vector of contact distances, and *α* is a matrix regularization weighting coefficient. The application of these impulses returns cells to mechanical equilibrium while also minimizing cell displacement.

*T6SS firing and costs*: T6SS+ cells can fire toxin-laden needles of length *L*_needle_ outwards from points on their surface. Every timestep d*t*, a focal T6SS+ cell *i* may fire *N*_firings,*i*_ ≥ 0 times. The number and spatial orientations of firings depend on the phenotype of the focal cell (Supplementary Table 1). If the focal cell is a Random-firing (R-type) strategist, *N*_firings,*i*_ is drawn from a Poisson distribution with mean *k*_fire_; these needles emanate from randomly chosen points on the focal cell’s surface (Supplementary Fig. 1B). For retaliatory TFT-type strategists, needles instead emanate from surface points at which the focal cell was struck; *N*_firings,*i*_ is then the number hits sustained by the focal cell in a given timestep. Similarly, two-tits-for-tat (2TFT-type) strategists fire back twice for every hit they sustain. To reflect the material and energetic costs of T6SS carriage and use, T6SS+ cells reduce their growth rate to $k_{{\mathrm{grow}},i} = k_{{\mathrm{max}}}\left( {1 - c_{{\mathrm{Total}},i}} \right)$, where $c_{{\mathrm{Total}},i} = c_{{\mathrm{upfront}}} + c\left( {N_{{\mathrm{firings}},i}/{\mathrm{d}}t} \right)$. Here, *c*_upfront_ represents the cost of T6SS gene carriage, while the latter term reflects the pro rata running costs of T6SS firing.

*T6SS hit detection*: To determine whether a given firing event is successful, we run a two-step hit detection algorithm to determine (i) whether that needle intersected any other cell in the population, and if so, (ii) where on the target cell the needle struck (Supplementary Fig. 1B). Both checks involve standard methods in computational geometry^59^: (i) involves computing the shortest distance *d*_min_ between the needle and cell line segments; $d_{{\mathrm{min}}} < \,R - L_{{\mathrm{penetration}}}$ indicates contact between the needle and the cell, where *R* is the cell radius of the victim, and *L*_peneration_ a small tolerance factor. Test (ii) involves checking whether a needle vector passes through the cylindrical midsection of the cell, or through spheres of radius *R* placed at its poles; whichever intercept lies closest to the needle’s origin is logged as the entry point (Supplementary Fig. 1B, middle, yellow stars). Here, we show an example of a needle (red arrow) that intercepts only the cell midsection, and a second example (magenta arrow) intercepting both the left polar sphere and the midsection.

*T6SS intoxication*: Any cell struck by a T6SS needle fired from a non-clonemate cell becomes intoxicated (cells of the same genotype are assumed mutually immune). Cells respond to T6SS intoxication with a step-like dose response: once a cell’s cumulative translocation count reaches threshold *N*_hits_, that cell begins to lyse. *N*_hits_ therefore parameterizes both the potency of a given T6SS effector and the capacity of a victim cell to withstand it. Lysing cells die—and are immediately removed from the simulation—after a delay of 1/*k*_lysis_, where *k*_lysis_ is the victim cell lysis rate. Lysing cells do not grow or consume patch resources.

### Model parameterization

The parameters of our model (21 in total) are summarized in Supplementary Table 3. Numerical and mechanical parameters controlling cell mechanical interactions and movement were taken from previous publications^27,39,40^. Where possible, parameters governing T6SS firing and response were estimated directly from previous experimental observations of T6SS competition^25,44^. We used a lower bound of *N*_hits_ = 1 based on a previous study^44^. However, based on the observation that *P. aeruginosa* seems to withstand multiple T6SS hits before being killed^27^, we also considered larger values (*N*_hits_ = 2, 3, and ∞ in cases where target cells possess immunity to oncoming T6SS attacks). Throughout the study, we used the parameter values *c* = 0.001, *c*_upfront_ = 0.05; we chose these values because they made the optimal firing rate in R vs. U competitions roughly consistent with the firing rates observed in random-firing bacteria like *V. cholerae* and *A. baylyi* (~50–100 firings cell^−1^ h^−1^)^27,44^. Since it was not possible to glean cost parameter values directly from experiments, we instead performed broad parameter sweeps to test the effects of different costs.

### Game theory

As in previous studies^49,60^, we use the logic of game theory and adaptive dynamics^47^ to determine whether a focal strategy (U, R, TFT, or 2TFT) could evolve from a given bacterial metapopulation, subject to different scales of competition. This method uses short-term competition outcomes to infer the evolutionary fate of a rare, novel strategy in a metapopulation where a different “resident” strategy predominates. If the novel strategist can reproduce faster than the resident strategist even when rare, its frequency in the metapopulation will increase, until eventually it supplants the resident. For example, to test whether an R strategist can invade a population of U strategists, we compare their effective fitnesses where one is rare, and the other common. For R to invade U, we require

1 $$W_{{\mathrm{rel}}}\left( {{\mathrm{rare}}\;{\mathrm{R}}|{\mathrm{common}}\;{\mathrm{U}}} \right) \, > \, 1,\quad \quad \,W_{{\mathrm{rel}}}\left( {{\mathrm{common}}\;{\mathrm{R}}|{\mathrm{rare}}\;{\mathrm{U}}} \right) \, \ge 1,$$

where *W*_rel_(X∣Y) is the relative fitness of X against Y. The first inequality specifies that R can invade U from rarity; the second checks that R is resistant to re-invasion by U once R becomes common. The definition of relative fitness *W*_rel_ depends upon the spatial scale of competition within the metapopulation. If competition is localized, then R competes primarily with nearby residents. Here, invasion is predicted simply from the ratio of strategists’ fitnesses within a spatial patch: R invades U provided that

2 $$\frac{{\omega _{\mathrm{R}}({\mathrm{R}}|{\mathrm{U}})}}{{\omega _{\mathrm{U}}({\mathrm{R}}|{\mathrm{U}})}} \, > \, 1,$$

where $\omega _{\mathrm{X}}({\mathrm{X}}|{\mathrm{Y}})$ is the fitness of strategist X in competition with strategist Y, with *ω*_X_ defined as $\omega _{\mathrm{X}} = {\mathrm{log}}_{\mathrm{e}}\left( {{\Sigma} V_{\mathrm{X}}(t_{{\mathrm{end}}})/{\Sigma} V_{\mathrm{X}}(t_{{\mathrm{start}}})} \right)$. Alternatively, competition may occur on much greater spatial scales, such that R must also compete with U strategists in other patches in the metapopulation. Assuming that R is initially rare, its encounters will predominately be with resident strategists, so its effective fitness is its reproductive capacity when in competition with U. Meanwhile, residents will encounter the novel strategy only rarely, and so will have an effective fitness based on reproduction when in competition with other residents. For R to invade U under these conditions, we require

3 $$\frac{{\omega _{\mathrm{R}}({\mathrm{R}}|{\mathrm{U}})}}{{\omega _{\mathrm{U}}({\mathrm{U}}|{\mathrm{U}})}} \, > \, 1,\quad\frac{{\omega _{\mathrm{R}}({\mathrm{R}}|{\mathrm{R}})}}{{\omega _{\mathrm{U}}({\mathrm{R}}|{\mathrm{U}})}} \, \ge \, 1.$$

We refer to these two sets of inequalities as local and global invasion constraints, respectively. To create the 1-D invasion plots shown in Fig. 1c, we computed mean values of $\omega _{\mathrm{R}}({\mathrm{R}}|{\mathrm{R}})$, $\omega _{\mathrm{U}}({\mathrm{U}}|{\mathrm{U}}),\quad \omega _{\mathrm{R}}({\mathrm{R}}|{\mathrm{U}})$, and $\omega _{\mathrm{U}}({\mathrm{R}}|{\mathrm{U}})$ for the R-strategist firing rates *k*_fire_ shown in Fig. 1b, linearly interpolating one additional value between each pair of adjacent data points. We then classified each firing rate according to which of the local and global invasion constraints held true. We used the same methodology for other pairs of strategists (replacing U with TFT, 2TFT or a second R strategist; cf. Supplementary Fig. 3). For global invasion analyses of R1 vs. R2 competition (Fig. 1e), we have the special case that the two global invasion constraints are equivalent (i.e., R1 invading R2 precludes R2 invading R1). Here, both strategists are characterized by their own independent firing rates *k*_fire,R1_,*k*_fire,R2_, and so invasion outcome is summarized by the 2-D color map,

4 $$I_{{\mathrm{inv}}}^{{\mathrm{global}}}\left( {k_{{\mathrm{fire}},{\mathrm{R}}1},\,k_{{\mathrm{fire}},{\mathrm{R}}2}} \right) = \omega _{{\mathrm{R}}2}({\mathrm{R}}2|{\mathrm{R}}1)/\omega _{{\mathrm{R}}1}({\mathrm{R}}1|{\mathrm{R}}1).$$

The corresponding invasion index for local competition scales (Fig. 1d) is

5 $$I_{{\mathrm{inv}}}^{{\mathrm{local}}}\left( {k_{{\mathrm{fire}},{\mathrm{R}}1},\,k_{{\mathrm{fire}},{\mathrm{R}}2}} \right) = \omega _{{\mathrm{R}}2}({\mathrm{R}}2|{\mathrm{R}}1)/\omega _{{\mathrm{R}}1}({\mathrm{R}}2|{\mathrm{R}}1).$$

### Incorporating mutual immunity

To test the effects of allowing for mutual immunity between T6SS+ strains, we re-ran competition simulations as before, except with T6SS attacks having no effect on T6SS+ target cells. We then expanded our definition of a strategist’s fitness, *ω*_X_, to be a weighted sum of its fitness against a competitor with (Y′) or without (Y) mutual immunity, $\omega _{\mathrm{X}}({\mathrm{Y}}) = p_{\mathrm{s}}\omega _{\mathrm{X}}({\mathrm{X}}|{\mathrm{Y}}^\prime ) + (1 - p_{\mathrm{s}})\omega _{\mathrm{X}}({\mathrm{X}}|{\mathrm{Y}})$. Note that we assume that T6SS carriage is a prerequisite for immunity, so unarmed (U) strategists are never immune and always have *p*_s_ = 0.

We then used this updated definition to recompute global invasion indices, now as a function both of firing rate *k*_fire,R_ and of the weighting parameter *p*_s_. The updated invasion indices are as follows: for R vs. U, we have

6 $$I_{{\mathrm{inv}},1}^{{\mathrm{global}}}\left( {k_{{\mathrm{fire}},{\mathrm{R}}}} \right) = \frac{{\omega _{\mathrm{R}}({\mathrm{R}}|{\mathrm{U}})}}{{\omega _{\mathrm{U}}({\mathrm{U}}|{\mathrm{U}})}} \, > \, 1,$$

7 $$I_{{\mathrm{inv}},2}^{{\mathrm{global}}}\left( {k_{{\mathrm{fire}},{\mathrm{R}}},\,p_{\mathrm{s}}} \right) = \frac{{\omega _{\mathrm{U}}({\mathrm{R}}|{\mathrm{U}})}}{{p_{\mathrm{s}}\omega _{\mathrm{R}}({\mathrm{R}}|{\mathrm{R}}^\prime ) + (1 - p_{\mathrm{s}})\omega _{\mathrm{R}}({\mathrm{R}}|{\mathrm{R}})}} \, \le \, 1,$$

and for R vs. Y = TFT or 2TFT, we have

8 $$I_{{\mathrm{inv,}}1}^{{\mathrm{global}}}\left( {k_{{\mathrm{fire,R}}},p_{\mathrm{s}}} \right) = \frac{{p_s\omega _{\mathrm{R}}({\mathrm{R}}|{\mathrm{Y}}^\prime ) + (1 - p_{\mathrm{s}})\omega _{\mathrm{R}}({\mathrm{R}}|{\mathrm{Y}})}}{{\omega _{\mathrm{Y}}({\mathrm{Y}}|{\mathrm{Y}})}} \, > \, 1,$$

9 $$I_{{\mathrm{inv}},2}^{{\mathrm{global}}}\left( {k_{{\mathrm{fire}},{\mathrm{R}}},p_{\mathrm{s}}} \right) = \frac{{p_{\mathrm{s}}\omega _{{\mathrm{Y}}^\prime }({\mathrm{R}}|{\mathrm{Y}}^\prime ) + (1 - p_{\mathrm{s}})\omega _{\mathrm{Y}}({\mathrm{R}}|{\mathrm{Y}})}}{{p_{\mathrm{s}}\omega _{\mathrm{R}}({\mathrm{R}}|{\mathrm{R}}^\prime ) + (1 - p_{\mathrm{s}})\omega _{\mathrm{R}}({\mathrm{R}}|{\mathrm{R}})}}\, \le \, 1.$$

We interpreted invasion indices as previously: for each strategist pairing, the inequalities $I_{{\mathrm{inv}},1}^{{\mathrm{global}}}\, > \, 1$ and $I_{{\mathrm{inv}},2}^{{\mathrm{global}}} \le 1$, respectively, test whether a focal strategist can invade a resident metapopulation from rarity, and resist re-invasion once established. For instance, for R vs. U, $I_{{\mathrm{inv}},1}^{{\mathrm{global}}} \,< \,1$ and $I_{{\mathrm{inv}},2}^{{\mathrm{global}}} \,> \, 1$ would indicate a circumstance in which U is able to invade R and not vice versa. In this way, we used invasion index traces to compute and generate stacks of pairwise invasion plots (Supplementary Fig. 8). These plots were colored and annotated according to invasion index values, using the key shown.

### Incorporating cheaters

To examine the effects of cheater emergence in our pairwise competition system, we created a new strategist (R_c_), representing a cheater strain emerging from a T6SS+ parent (which could be R, or TFT, or 2TFT). R_c_ possesses the same T6SS genes as its parent (conferring possible immunity to other T6SS+ strains, in exchange for the growth cost of T6SS gene carriage *c*_upfront_), but never invests in any T6SS activity ($k_{{\mathrm{fire}},{\mathrm{R}}_{\mathrm{c}}}$ = 0). We then computed pairwise (global) invasion indices for R_c_ vs. every other strain, in the following groupings: R vs. U vs. R_c_, R vs. TFT vs. R_c_, and R vs. 2TFT vs. R_c_. We determined invasion behavior using the same invasion index system as before, again considering both cases with mutual vulnerability and mutual immunity. This introduced four additional invasion indices in each grouping, whose values indicate whether the cheater can invade (and avoid re-invasion by) the other two strategists in each case. Thus, the invasion behavior within each grouping is summarized by the values of six invasion indices.

### Incorporating reduced in-patch relatedness

To incorporate the effects of increased within-patch relatedness, we created a second weighting scheme in which an R-strategist’s effective fitness *ω*_R_ is a weighted sum of (i) fitness when growing alone (i.e., paired with itself), and (ii) fitness growing alongside a competitor Y. Note that being paired with self implies mutual immunity to T6SS attacks, since by assumption all clonemates share identical T6SS effector and immunity genes. Thus, we have

10 $$\omega _{\mathrm{R}}({\mathrm{Y}}) = I\omega _{\mathrm{R}}({\mathrm{R}}|{\mathrm{R}}^\prime ) + (1 - I)\omega _{\mathrm{R}}({\mathrm{R}}|{\mathrm{Y}}),$$

where *I* is a weight parameter representing the probability of perfect strategist segregation, and where $\omega _{\mathrm{R}}\left( {{\mathrm{R}}|{\mathrm{R}}^{\prime}} \right)$ represents R’s fitness when grown with mutually immune clonemates. In this regime, the local invasion constraint then becomes

11 $$I_{{\mathrm{inv}}}^{{\mathrm{local}}}\left( {k_{{\mathrm{fire}},{\mathrm{R}}},I} \right) = \frac{{I\omega _{\mathrm{R}}({\mathrm{R}}|{\mathrm{R}}^\prime ) + (1 - I)\omega _{\mathrm{R}}({\mathrm{R}}|{\mathrm{Y}})}}{{I\omega _{\mathrm{Y}}({\mathrm{Y}}|{\mathrm{Y}}^\prime ) + (1 - I)\omega _{\mathrm{Y}}({\mathrm{R}}|{\mathrm{Y}})}} \, > \, 1,$$

for Y = U, TFT, or 2TFT. For global invasion, the (common) resident strategist will statistically only encounter other competitors sharing that strategy. Here, within-patch relatedness controls whether those resident competitors have the same or different effector/immunity sets, that is, the probability of mutual immunity:

12 $$\omega _{{\mathrm{Y}}\!,{\mathrm{common}}} = I\omega _{\mathrm{Y}}({\mathrm{Y}}|{\mathrm{Y}}^\prime ) + (1 - I)\omega _{\mathrm{Y}}({\mathrm{Y}}|{\mathrm{Y}}).$$

Note that Y = U, TFT, or 2TFT undertake no T6SS firing when paired in this way; in these cases, this equation reduces to $\omega _{{\mathrm{Y}},{\mathrm{common}}} = \omega _{\mathrm{Y}}\left( {{\mathrm{Y|Y}}} \right).$ Therefore, our global invasion indices become

13 $$I_{{\mathrm{inv}},1}^{{\mathrm{global}}}\left( {k_{{\mathrm{fire}},{\mathrm{R}}},I} \right) = \frac{{I\omega _{\mathrm{R}}({\mathrm{R}}|{\mathrm{R}}^\prime ) + (1 - I)\omega _{\mathrm{R}}({\mathrm{R}}|{\mathrm{Y}})}}{{\omega _{\mathrm{Y}}({\mathrm{Y}}|{\mathrm{Y}})}} \, > \, 1,$$

14 $$I_{{\mathrm{inv}},2}^{{\mathrm{global}}}\left( {k_{{\mathrm{fire}},{\mathrm{R}}},I} \right) = \frac{{I\omega _{\mathrm{Y}}({\mathrm{Y}}|{\mathrm{Y}}^\prime ) + (1 - I)\omega _{\mathrm{Y}}({\mathrm{R}}|{\mathrm{Y}})}}{{I\omega _{\mathrm{R}}({\mathrm{R}}|{\mathrm{R}}^\prime ) + (1 - I)\omega _{\mathrm{R}}({\mathrm{R}}|{\mathrm{R}})}} \, \le \, 1,$$

for Y = U, TFT, or 2TFT. We used these indices to construct 1-D invasion plots (as in Figs. 1 and 2 and Supplementary Fig. 3) for *I* values of 0.01, 0.5, and 0.99, representing increasing levels of within-patch relatedness. These results are plotted in Supplementary Figs. 11 and 12.

### Computation and postprocessing

Agent-based model simulations were run on a 2017 Apple ® MacBook Pro laptop computer, with simulations distributed between an Intel ® 3.1 GHz quadcore i7-7920HQ CPU, an Intel ® HD 630 Graphics card, and an AMD Radeon Pro 560 Compute Engine. Simulation data were analyzed using custom Matlab ® scripts (version R2017a 9.2.0.556344), and visualized using the Paraview software (version 5.4.0)^61^.

### Bacterial strains and growth conditions

*Pseudomonas aeruginosa* PAO1 *tssB-mNeonGreen*, *V. cholerae* 2740-80 *vipA-mCherry2*, and *V. cholerae* 2740-80 *vipA-mCherry2 Δhcp1 Δhcp2* were inoculated from Luria broth (LB) agar plates and grown aerobically at 37 °C in LB to an OD600 of 1 (~3 h). One milliliter of each day culture was then pelleted at 11,000 × *g* for 1.5 min and resuspended in LB to reach OD600 of 10. *Pseudomonas aeruginosa* PAO1 *tssB-mNeonGreen* was mixed with *V. cholerae* 2740-80 *vipA-mCherry2* or *V. cholerae* 2740-80 *vipA-mCherry2 Δhcp1 Δhcp2* in a 1:5 ratio (10–50 µl). Both mixtures (1.5 µl) were spotted on a pad of 1% agarose in 1/3 LB and 2/3 phosphate-buffered saline). The pad was covered with a glass coverslip and incubated for 30 min at 30 °C before imaging.

### Fluorescence microscopy

For live-cell fluorescence microscopy, the same equipment was used as described previously^62,63^; a Nikon Ti-E inverted microscope with Perfect Focus System and a Plan Apo 1003 Oil Ph3 DM (NA 1.4) objective lens, a SPECTRA X light engine (Lumencore), and ET-GFP (Chroma #49002) and ET-mCherry (Chroma #49008) filter sets to excite and filter fluorescence. Exposure time was set to 150 ms and LED powers to 20%. Images were recorded with a sCMOS camera pco.edge 4.2 (PCO, Germany; 65-nm pixel size) and VisiView software (version 4.4.0.10, Visitron Systems, Germany). Imaging was carried out at 30 °C and 95% humidity controlled by an Okolab T-unit (Okolab) and images were collected every 2 s for 5 min. The imaging experiments were performed in two biological replicates.

### Image analysis

Image analysis and manipulation was carried out with Fiji^64^. Contrasts were set equally for a set of compared images. Intensity of GFP and mCherry channels was corrected with the simple ratio bleach correction function. Numbers of *P. aeruginosa* PAO1 *tssB-mNeonGreen* cells in contact with *V. cholerae* 2740-80 *vipA-mCherry2* or *V. cholerae* 2740-80 *vipA-mCherry2 Δhcp1 Δhcp2* cells were counted based on the phase contrast and GFP channel. The number of T6SS structures per cell in *P. aeruginosa* PAO1 *tssB-mNeonGreen* was counted in the maximum intensity projection image of the GFP channel. Only T6SS structures of cells in contact with *V. cholerae* were counted. To quantify the number of repeated T6SS assemblies in kymograms, the reslice function was used. Only repeated T6SS assemblies directed towards *V. cholerae* cells were analyzed. Kymograms of *V. cholerae* were used to calculate the time without new T6SS after contraction (2 s pixel^−1^). Only T6SS assemblies directed towards *P. aeruginosa* cells were included in the analysis. All quantifications were performed manually. GraphPad Prism7 was used to display the histogram of repeated T6SS assemblies. The number of cells analyzed, averages with standard deviations, and medians are given in the figure legend.

### Statistical analyses

Unless indicated otherwise, the number of simulation replicates is five for each parameter combination shown. Two biological replicates were used in all experiments. For comparative statistics (Fig. 3b, d), we used a two-sample, two-sided *t* test, assuming data normality. No adjustments were made for multiple comparisons. To estimate effect sizes, we used Cohen’s *d* measure, $d = \frac{{\left( {\mu _1 - \mu _2} \right)}}{{\sqrt {({\mathrm{SD}}_1^2 + {\mathrm{SD}}_2^2)/2} }}$, where *μ*_*i*_ and SD_*i*_ are, respectively, data means and standard deviations for each sample *i*. All statistical calculations were performed in Matlab ® (version R2017a 9.2.0.556344).

### Reporting summary

Further information on research design is available in the Nature Research Reporting Summary linked to this article.

## Supplementary information

Supplementary Information

Reporting Summary

Description of Additional Supplementary Files

Supplementary Movie 1

Supplementary Movie 2

Supplementary Movie 3

Supplementary Movie 4

Supplementary Movie 5

Supplementary Movie 6

Supplementary Movie 7

Supplementary Movie 8

## Data Availability

Source data are provided with this paper.

## Source data

Source Data

## Code availability

Our model software is based on CellModeller, a GPU-compatible Python/OpenCL modeling framework^39,40^. Our source code is available to download from Github (https://github.com/WilliamPJSmith/CellModeller).
