# Supplementary material for: The evolution of tit-for-tat in bacteria via the type VI secretion system
Source: Nat Commun. 2020 Oct 26;11:5395. doi: 10.1038/s41467-020-19017-z (PMC7589516; doi:10.1038/s41467-020-19017-z)
Supplement: Supplementary file 1 — Supplementary Information [file 41467_2020_19017_MOESM1_ESM.pdf]

Supplementary Information

The Evolution of Tit-for-Tat in Bacteria via the Type VI Secretion System

Smith et al.

Author Information

William P. J. Smith[1,2] *, Maj Brodmann[3], Daniel Unterweger[4,5], Yohan Davit[6], Laurie E. Comstock[7], Marek Basler[3], and Kevin R. Foster[1,2] *

1. Department of Zoology, University of Oxford, OX1 3SZ, UNITED KINGDOM
2. Department of Biochemistry, University of Oxford, OX1 3QU, UNITED KINGDOM
3. Biozentrum, University of Basel, Klingelbergstrasse 50/70, CH-4056 Basel, SWITZERLAND
4. Institute for Experimental Medicine, Kiel University, 24105 Kiel, GERMANY
5. Max-Planck Institute for Evolutionary Biology, 24306 Plön, GERMANY
6. Institut de Mécanique des Fluides de Toulouse, CNRS and Université de Toulouse, 31400 Toulouse, FRANCE
7. Division of Infectious Diseases, Brigham and Women's Hospital, Boston, MA 02115, UNITED STATES

* Corresponding author. Email: william.smith@zoo.ox.ac.uk, kevin.foster@zoo.ox.ac.uk

Supplementary Methods

Assessing the impact of T6SS toxin diversity

In our analyses, we assumed that the toxin and immunity genes of T6SS+ strategists differ, such that different strains and strategists are always vulnerable to each other's attacks. Generally, T6SS toxin repertoires seem to be diverse and highly variable within and between species, and so we anticipate that this assumption will generally hold (*1*, *2*). However, exceptions are clearly possible, and so we explored cases where effector toxin diversity is low, such that a focal strain frequently encounters competitors that share identical sets of toxin and immunity genes.

Specifically, we re-ran competition simulations with T6SS+ strategists (R, TFT or 2TFT) possessing mutual immunity, instead of mutual vulnerability. Then, for each competition scenario, we redefined the fitness of a focal strain X, ω_X, as the weighted mean of these two cases,

$$\omega_X(Y) = p_s\omega_X(X|\,Y') + (1 - p_s)\omega_X(X|\,Y), \quad (1)$$

where Y' and Y represent competitors with either the same or different toxin and immunity genes as X, respectively. The weight parameter p_S, representing the likelihood of mutual immunity, can then be increased to test the effects of (e.g.) reducing the diversity of T6SS toxins within a metapopulation, such that randomly-paired T6SS+ competitors are more likely to be mutually immune.

Supplementary Figure 8 shows the effects of increasing the likelihood of mutual immunity (p_S) for our R-U, R-TFT and R-2TFT global invasion analyses. We plot vertical stacks of 1-D invasion diagrams, color-coded as in Figures 1 and 2, for increasing values of p_S. These diagrams show variation in p_S has a minimal effect on the model's predictions. In unilateral competitions between U and R (Supplementary Figure 8, top row), high p_S increases R's fitness when common, and so makes it marginally more difficult to invade by U at lower cell densities (Supplementary Figure 8, top row). For R vs TFT, 2TFT competitions, high p_S tends to disfavor whichever competitor is the superior killer, since mutual immunity deprives it of 'kills' it would otherwise benefit from (Supplementary Figure 8, middle and bottom rows). Thus, increasing p_S expands the range of conditions for which TFT invades R. However, this effect is insufficient to allow R to robustly invade 2TFT, which retains its supremacy.

The fate of T6SS cheater strategists

Another possibility to examine is the potential emergence of constitutive cheater strategists: T6SS+ bacteria which do not engage in T6SS firing, but which still benefit indirectly from the killing effects of T6SS-active strategists. For instance, such a cheater might arise via mutation in a population of random T6SS attackers, and then displace its parent (whose attacks it retains immunity to, while avoiding the growth costs of T6SS use). To examine the effects of

incorporating cheaters in our invasion analyses, we created a new strategist "R_c" that pays for T6SS carriage (and potential immunity) but which never fires the weapon ($k_{fire} = 0$; see Methods). We detail the resulting effects below but, critically, cheaters have little impact so long as toxin diversity is high (low p_S), as expected from the biology of T6SS (*1*, *2*). This result occurs because, with high toxin diversity, cheaters are simply killed by competitors as they have the wrong immunity protein for their competitor.

We re-ran our U vs R, R vs TFT and R vs 2TFT pairwise invasion analyses, this time also checking whether the cheater R_c could invade (or be invaded by) the other competing strategists, from an initial state of rarity. The outcomes of this 3-way invasion analysis are shown in Supplementary Figures 9 and 10, which extend the results of Supplementary Figure 8 to incorporate the cheater (again, for different weapon firing rates, cell densities and mutual immunity probabilities).

As discussed above, the impact of introducing the cheater depends upon the probability of mutual immunity, p_S. For low values of this parameter (high toxin diversity), cheater strategists are generally unsuccessful: since their T6SS genes rarely confer protection against random attackers, cheaters are effectively a slower-growing version of U, and will likewise be invaded by R (Supplementary Figures 9 and 10). For higher p_S values, cheaters can gain the benefits of widespread immunity, and can affect invasion outcomes. Specifically, they can act as invasion intermediaries, facilitating an invasion that would otherwise not be possible (for instance, in Supplementary Figure 9, zones labelled '4' show U invading R in a manner impossible without R_c). Additionally, they can facilitate non-transitive cycles of invasion, resembling the 'rock-paper-scissors' dynamics reported previously (e.g. zones '5' in Supplementary Figure 9; zones '16' in Supplementary Figure 10). However, cheaters generally have less of an impact on retaliator strategies (Supplementary Figure 10) than on random attackers (Supplementary Figure 9), since cheaters do not provoke the former into making potentially wasteful attacks. Moreover, they do not affect the supremacy of 2TFT over TFT and R.

The effects of within-patch relatedness

Another of our model's assumptions is within-patch relatedness is fixed, with competing strategists always mixing in a set (1:1) ratio when patches are seeded. Clearly however, it is possible for other mixing ratios to occur – with the extreme case being that a competitor only encounters its own clonemates within a patch (1:0 ratio). To account for variation in within-patch relatedness, we considered an additional fitness definition equivalent to an I-weighted average of these two extrema ($I = 0$ giving perfect assortment and $I = 1$ giving perfect segregation; see Methods). We then re-ran our local and global invasion tests (Supplementary Figure 3) for different weight values I; these results are shown in Supplementary Figures 11 (R vs U) and 12 (R vs TFT, 2TFT). We found that increasing I tended to disfavor whichever strategist was the superior killer, since that strategist thereby became segregated from competitors that it would otherwise kill. Increasing relatedness via I decreased the optimal rate

of firing, $k^{*}_{fire,R}$, against U (Supplementary Figure 11A, and likewise made U more robust at resisting invasion by R (Supplementary Figure 11B). Similarly, it made TFT better at invading R – but again, this effect was insufficient to enable R to invade 2TFT (Supplementary Figure 12), and 2TFT remained superior to TFT for all density conditions tested.

Supplementary Tables

T6SS phenotype	$N_{firings}$	Growth rate	Firing pattern
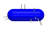 U = Unarmed	0	k_{max}	None
R = Random attacker	$k_{fire,R}\ dt$	$k_{max}(1 - c_{upfront} - cN_{firings,i} / dt)$	Random
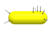 TFT = Tit-for-tat	1 x num. incident attacks	$k_{grow}(1 - c_{upfront} - cN_{firings,i} / dt)$	From incident attack points
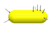 2TFT = 2-Tits-for-tat	2 x num. incident attacks	$k_{grow}(1 - c_{upfront} - cN_{firings} / dt)$	From incident attack points

Supplementary Table 1: T6SS strategists used in the agent-based model.

Category	Variable	Symbol	Units
Geometric	Position vector	$\mathbf{p}_i = (p_x, p_y, p_z)_i$	μm
	Orientation unit vector	$\mathbf{a}_i = (a_x, a_y, a_z)_i$	-
	Segment length	L_i	μm
	Volume	$V_i = 4\pi R^3/3 + \pi L_i R^2$	μm^3
T6SS	Specific growth rate	$k_{grow,i} = k_{grow,max}(1-c_{total,i})$	h^{-1}
	Total cost	$c_{total,i} = c_{upfront} + c\ (k_{fire,i})$	%
	Firing rate	$k_{fire,i} = N_{firings,i}(t) / dt$	Firings h^{-1}
	Firings this timestep	$N_{firings,i}$	Firings
	Cumulative hits	$N_{hits,i}$	Hits
Genetic	Cell genotype	U, R, TFT or 2TFT	-

Supplementary Table 2: Table of agent-based model variables.

Category	Parameter	Symbol	Value	Units	Source
Domain	Inoculum zone size	$D_{homeland}$	100	µm	This study
	Inoculum population	N_{cells}	20-400	cells	This study
	Domain carrying capacity	E_0	10,000 V_0	-	This study
Cell-based	Max. growth rate	k_{max}	1.0	h^{-1}	(1)
	Upfront T6SS cost	$c_{upfront}$	5	%	This study
	Pro rata T6SS cost	c	0.05 -0.5	% per k_{fire}	This study
	T6SS firing rate	k_{fire}	0-250.0	firings $cell^{-1}$ h^{-1}	Estimated from (2)
	Lysis delay	k_{lysis}	0.8-8.0	h^{-1}	Estimated from (2)
	Lethal hit threshold	N_{hits}	1-3	-	This study
	Needle length	L_{needle}	0.5	µm	Estimated from (2)
	Min. needle penetration	$L_{penetration}$	10	nm	Estimated from membrane width
	Cell radius	R	0.5	µm	Estimated from (2)
	Cell volume at birth	V_0	1.16	$µm^3$	Estimated from (2)
	Division volume noise	$\eta_{division}$	9	%	(3)
	Division orientation noise	$\eta_{orientation}$	0.2	%	(3)
Numerical	Simulation timestep	dt	0.025	h	(1)
	Grid element size	h	10	µm	(3)
	CG absolute tolerance	ϵ_{CG}	0.001	-	(3)
	Max. contact iterations	$M_{Iter, max}$	8	-	(1)
Mechanical	Regularization weight	α	0.04	-	(3)
	Growth restriction factor	1 / ɣ	0.002	-	(3)

Supplementary Table 3: Table of agent-based model parameters.

Supplementary Figures

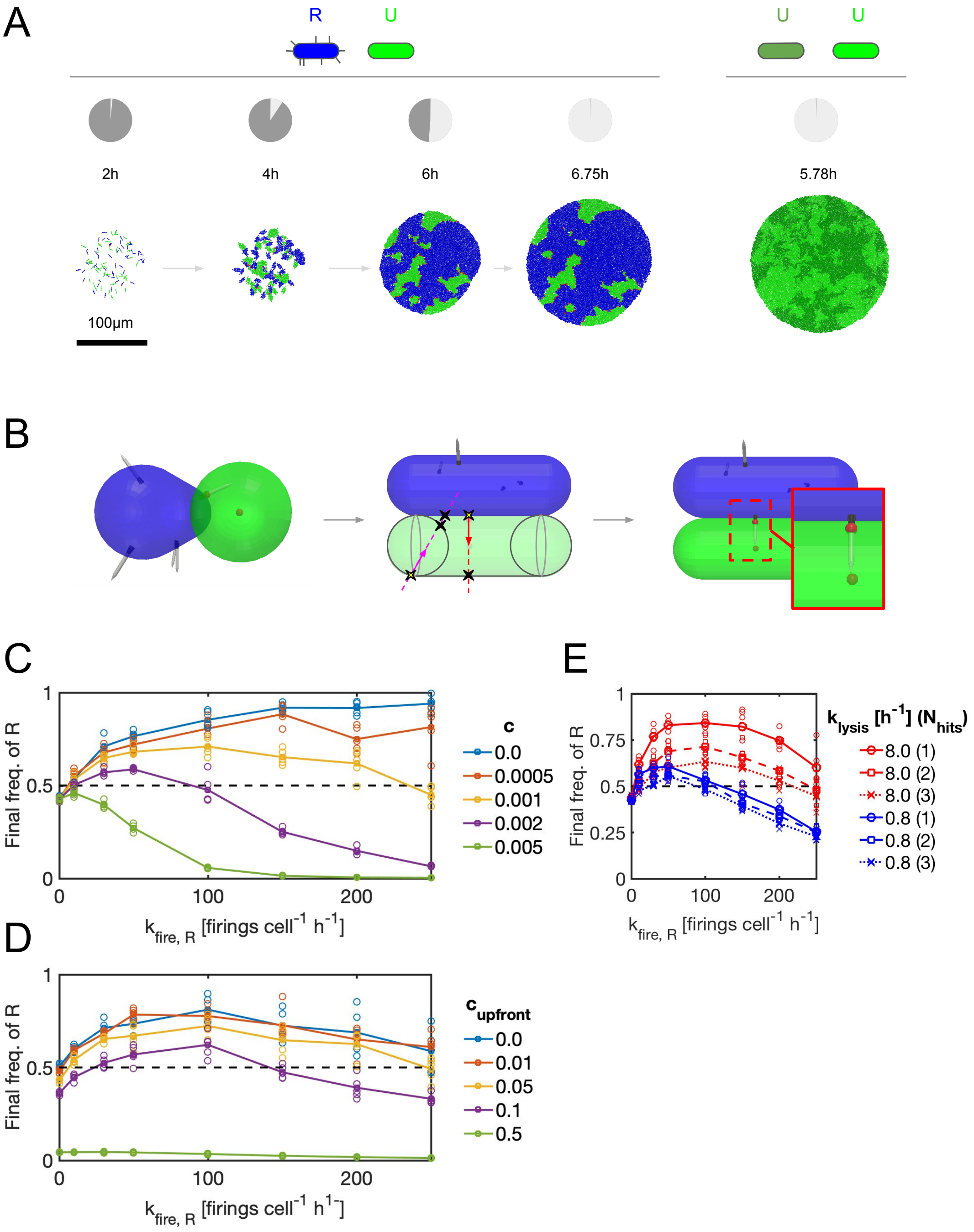

Supplementary Figure 1: Agent-based modelling of T6SS-mediated competition. (**A**) Simulation time-lapse showing competition between Random T6SS attackers (R, blue) and T6SS- Unarmed cells (U, green) in a resource-limited niche. Final state (6.95 h) compared with that for U vs. U competition (right, 5.78 h). Pie charts track resource depletion; simulation parameters $N_{\text{hits}} = 2$, $c = 0.001$, $k_{\text{fire,R}} = 50.0$ firings cell^{-1} h^{-1}. (**B**) Overview of hit detection system: each T6SS needle is checked for intersection with neighbor cells' midsections and polar spheres (middle). (**C,D,E**) Parameter sweeps for R vs. U competitions, plotting final R proportion against firing rates $k_{\text{fire,R}}$ for different pro rata cost factors c (C), carriage costs c_{upfront} (D), lysis rates k_{lysis} (E) and effector potencies N_{hits} (E). Data means (lines) and individual data points (circles) are shown. Parameters: $N_{\text{hits}} = 2$, $k_{\text{lysis}} = 8.0$ h^{-1}, $c_{\text{upfront}} = 0.05$ (C); $N_{\text{hits}} = 2$, $k_{\text{lysis}} = 8.0$ h^{-1}, $c = 0.001$ (D), $c = 0.001$, $c_{\text{upfront}} = 0.05$ (E). 5 replicates per case; initial cell density 50 vs. 50 cells throughout. Source data are provided as a Source Data file.

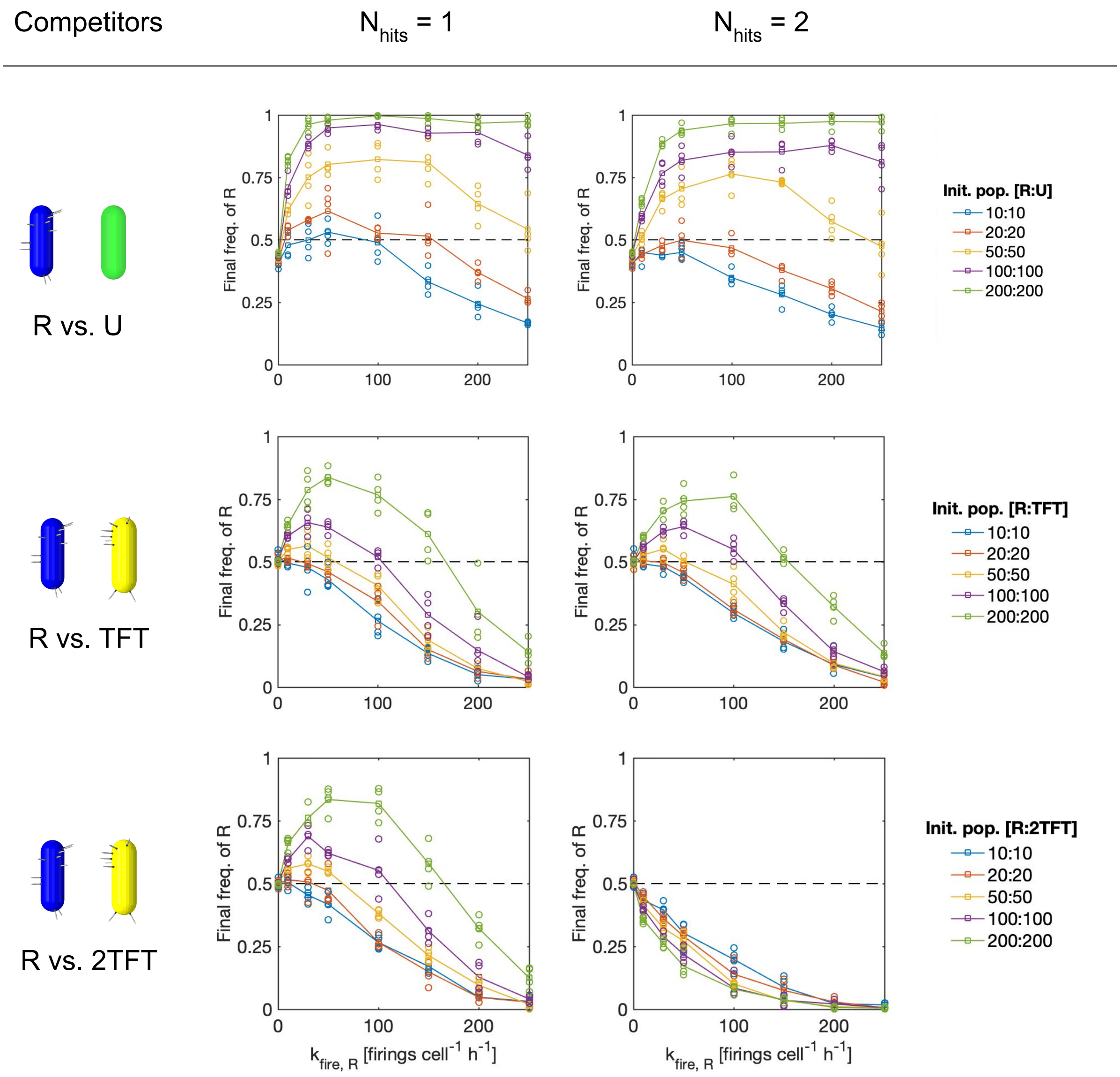

Supplementary Figure 2: Comparison of competition simulations between T6SS strategists. Repeats of $k_{\mathrm{fire,R}}$ vs. cell density parameter sweeps, showing competition outcomes for different competitors (rows) and T6SS effector potencies N_{hits} (columns). Data means (lines) and individual data points (circles) are shown. Panels analogous to those shown in Figures 1 and 2; N_{hits} = 2 cases repeated here for reference. k_{lysis} = 8.0 h^{-1}, c = 0.001 and 5 replicates per case throughout. Source data are provided as a Source Data file.

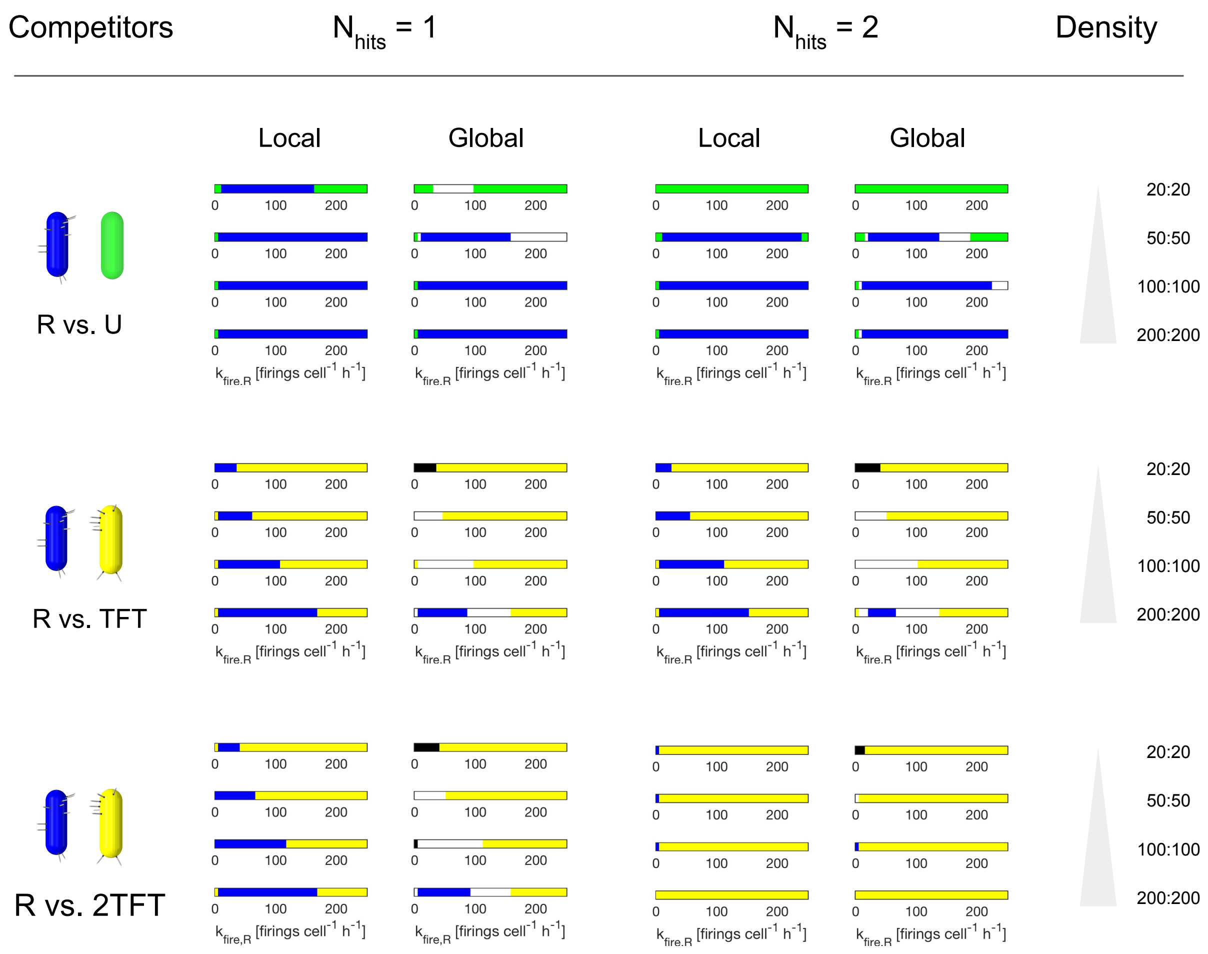

Supplementary Figure 3: Comparison of invasion analyses between T6SS strategists. 1-D invasion plots show outcomes of local and global invasion analyses, analogous to those shown in Figures 1 and 2, for different competitors (left column) intial cell densities (right column), and T6SS effector potencies (middle two columns). $N_{\text{hits}} = 2$ cases repeated here for reference; color legends as in Figures 1 and 2. $k_{\text{lysis}} = 8.0$ h^{-1}, $c = 0.001$ and 5 replicates per case throughout. Source data are provided as a Source Data file.

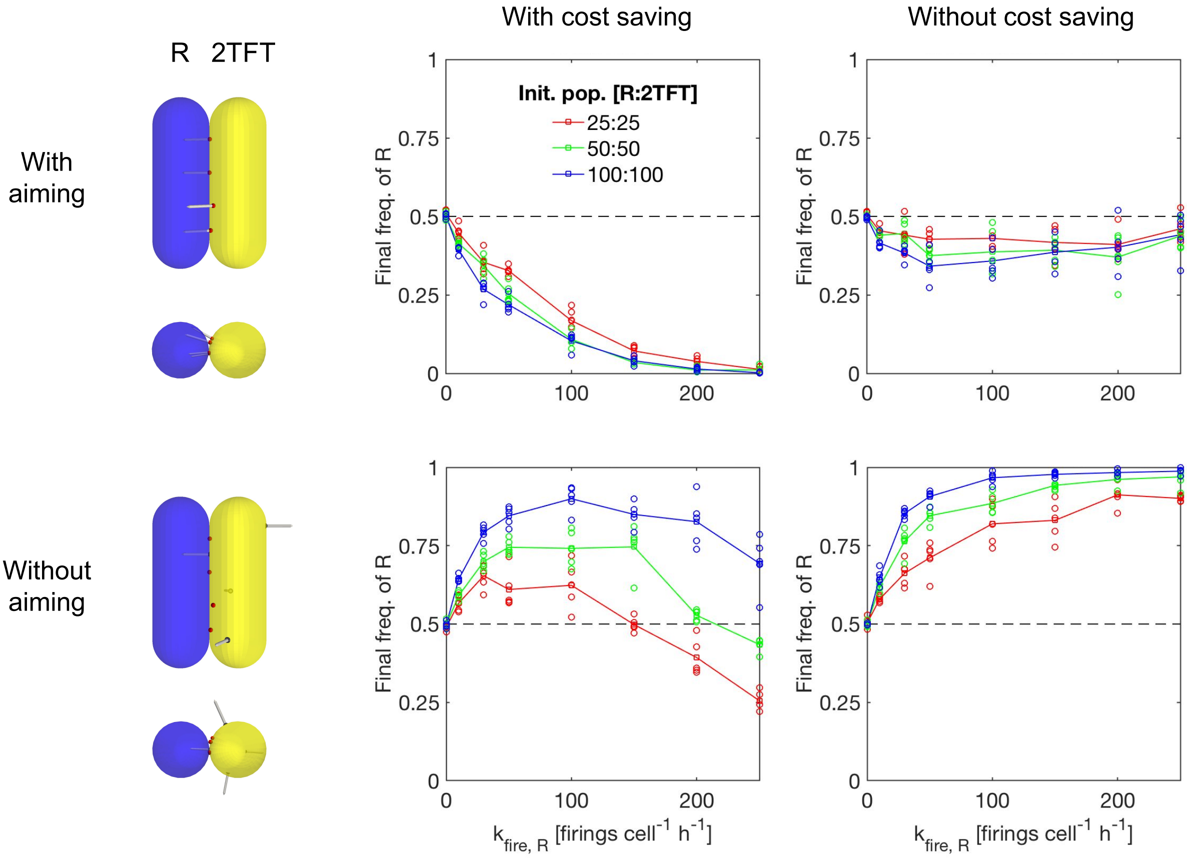

Supplementary Figure 4: Additional 2TFT knockout competitions. Repeats of R vs. 2TFT advantage knockout parameter sweeps from Figure 3, showing variation in competition outcome with initial cell density (see legend). Data means (lines) and individual data points (circles) are shown. $N_{hits} = 2, k_{lysis} = 8.0$ h^{-1}, $c = 0.001$ and 5 replicates per case throughout. Source data are provided as a Source Data file.

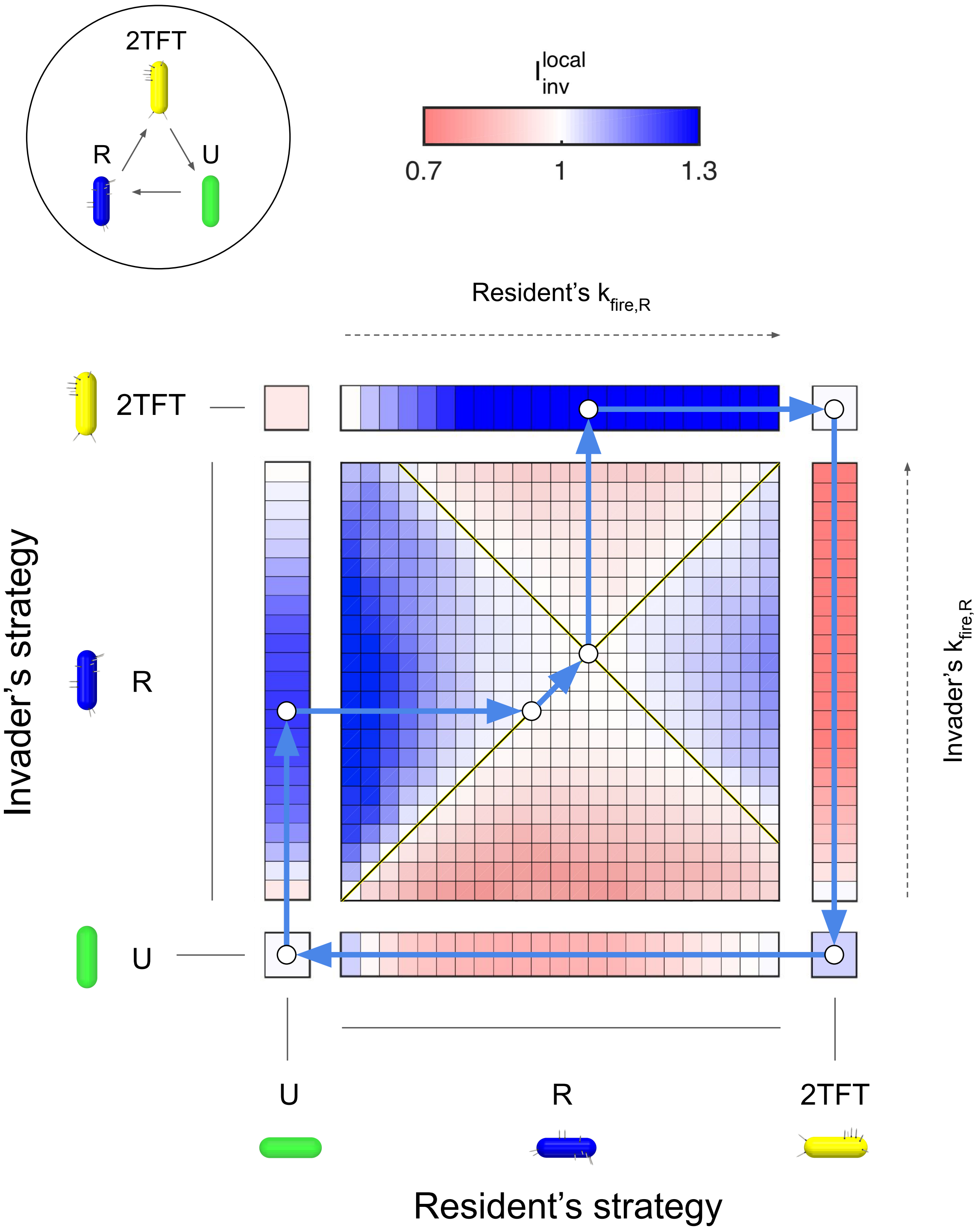

Supplementary Figure 5: Rock-paper-scissors dynamics between Unarmed, Random-Firing and 2-Tits-For-Tat strategists. Multi-strategy pairwise invasion plot showing non-transitive cycling between Unarmed (U), Random-firing (R) and 2-Tits-For-Tat (2TFT) strategies (blue arrows). Colormaps show values of invasion index for local competition scales (equivalent to relative invader fitness, see Methods). Simulation parameters: $N_{hits} = 2, c = 0.001$, initial cell density 50 vs. 50 cells. 5 simulation replicates per parameter combination tested. Source data are provided as a Source Data file.

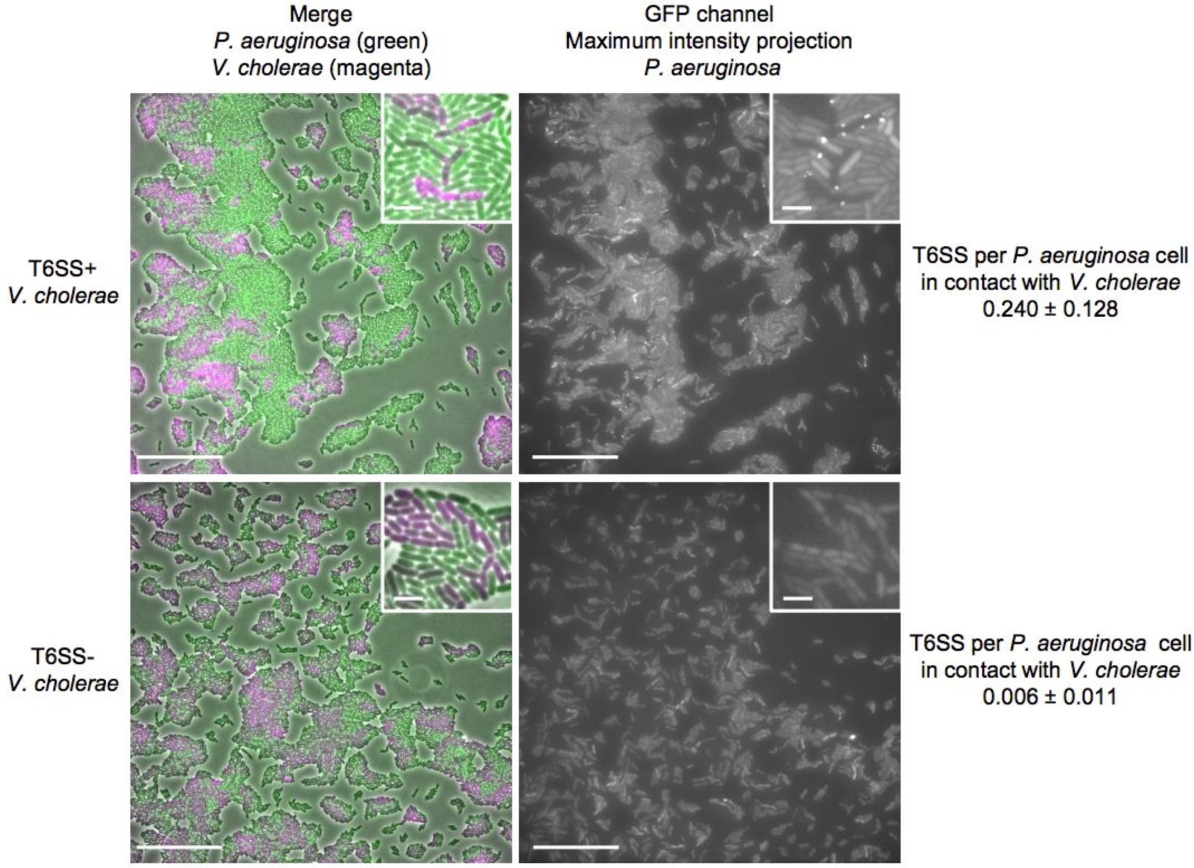

Supplementary Figure 6: Activation of *P. aeruginosa* T6SS by *V. cholerae* T6SS. Mixture of *P. aeruginosa* PAO1 *tssB-mNeonGreen* with T6SS+ *V. cholerae* 2740-80 *vipA-mCherry2* (top) or with T6SS- *V. cholerae* 2740-80 *vipA-mCherry2 Δhcp1 Δhcp2* (bottom). A merge of phase contrast, GFP and mCherry channels is shown (left) as well as the maximum intensity projection of the GFP channel with the accumulated T6SS events of *P. aeruginosa* PAO1 *tssBmNeonGreen* within 5 minutes (right). Large images have a field of view of 133.2 x 133.2 µm and the scale bars represent 30 µm. Small images are close ups and show a field of view of 13 x 13 µm and the scale bars represent 3 µm. T6SS structures per *P. aeruginosa* PAO1 *tssB-mNeongreen* cell in contact with either *V. cholerae* 2740-80 *vipA-mCherry2* (top) or *V. cholerae* 2740-80 *vipA-mCherry2 Δhcp1 Δhcp2* (bottom) is shown (average with standard deviation, n > 6500 cells, 2 biological replicates). Source data are provided as a Source Data file.

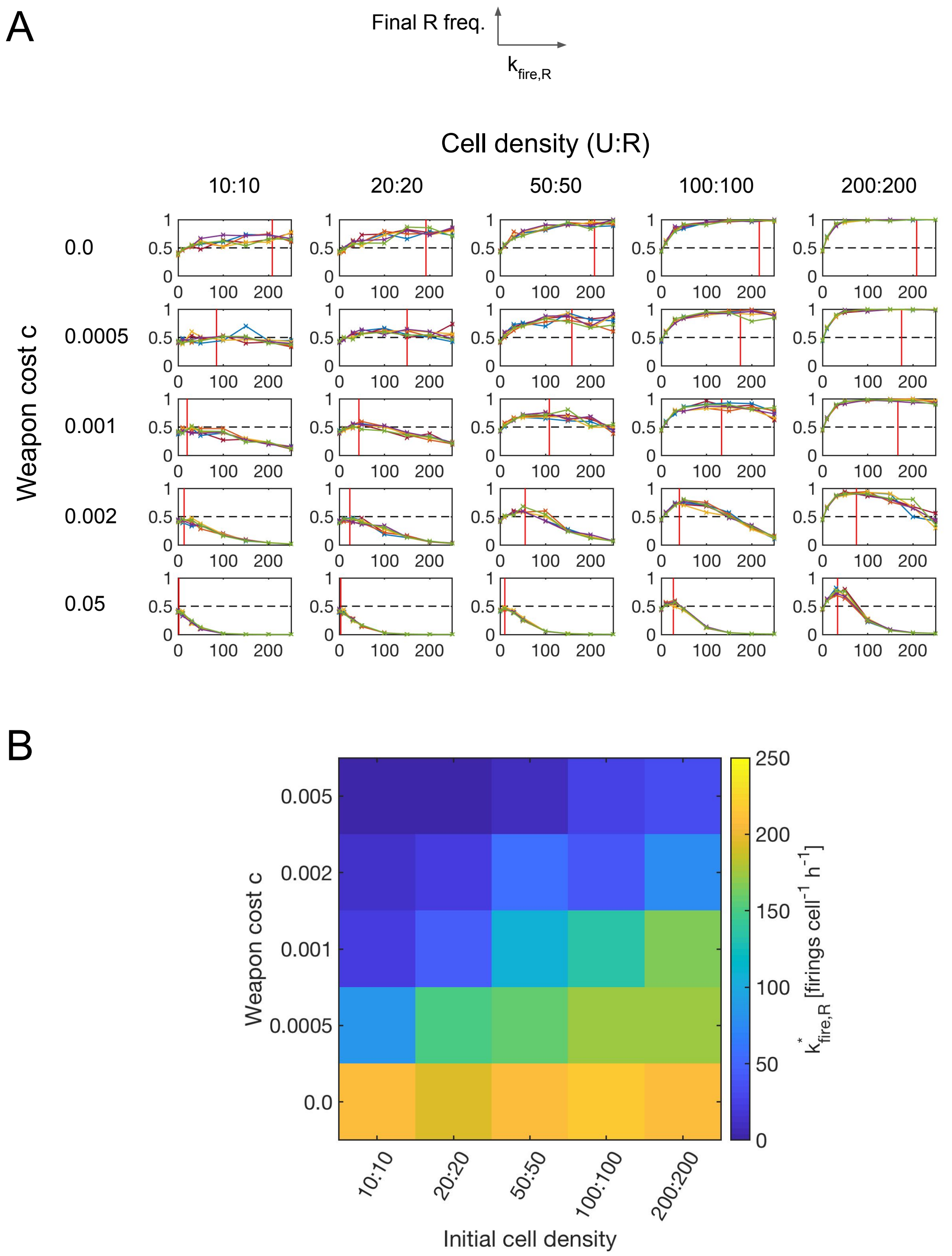

Supplementary Figure 7: High cell densities and low weapon costs maximize optimum T6SS firing rate. (**A**) Plots of final R cell frequency following competition with U, as a function of R strategist firing rate $k_{\mathrm{fire,R}}$, for different weapon cost parameters (c, rows) and initial cell densities (columns). In each plot, a set of 5 replicates are shown as individual traces; vertical red lines indicate the optimal firing rate ($k_{\mathrm{fire,R}}{}^{*}$) in each case (average of 5 replicates), defined as the $k_{\mathrm{fire,R}}$value that maximizes final R cell frequency. (**B**) Colormap summarizing optimum firing rates $k_{\mathrm{fire,R}}{}^{*}$ from A, as a function of weapon cost and initial cell density. Parameters: $k_{\mathrm{lysis}} = 8.0$ h^{-1}, $N_{\mathrm{hits}} = 2$. Source data are provided as a Source Data file.

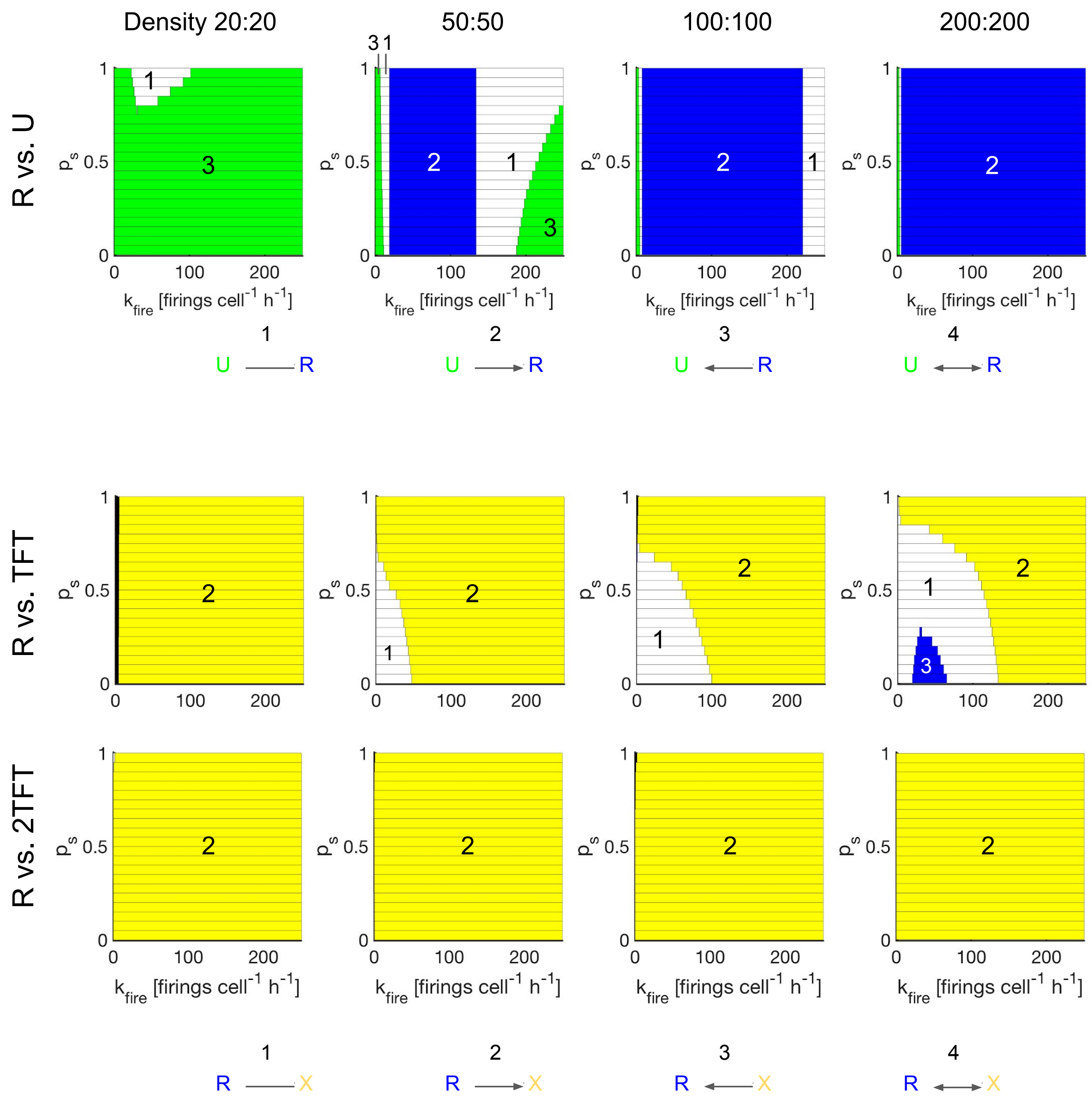

Supplementary Figure 8: Global invasion plots for variable T6SS firing rate k_{fire} and self-immunity probability p_s. Columns in the grid correspond to different cell densities tested (initial cell counts 20:20, 50:50, 100:100 and 200:200); rows depict competitions between different strategists. Each plot is color-coded, with colored zones numbered 1-4. The arrow diagrams below the plots depict the invasion behavior within each numbered zone. Arrows point towards strategist(s) that can invade competitors from rarity; depending on the conditions, cases with no invasion (1), unidirectional invasion (2,3) or mutual invasion (4) may be possible. Competitor 'X' corresponds to either TFT (middle row) or 2TFT (bottom row). Plots constructed using fitness values (mean of 5 replicates) for 8 k_{fire} values with 4 (U vs R; 160 simulations per plot) or 5 (R vs TFT, 2TFT, 200 simulations per plot) competitor pairings. Parameters: $k_{\mathrm{lysis}} = 8.0$ h^{-1}, $c = 0.001$, $N_{\mathrm{hits}} = 2$. Source data are provided as a Source Data file.

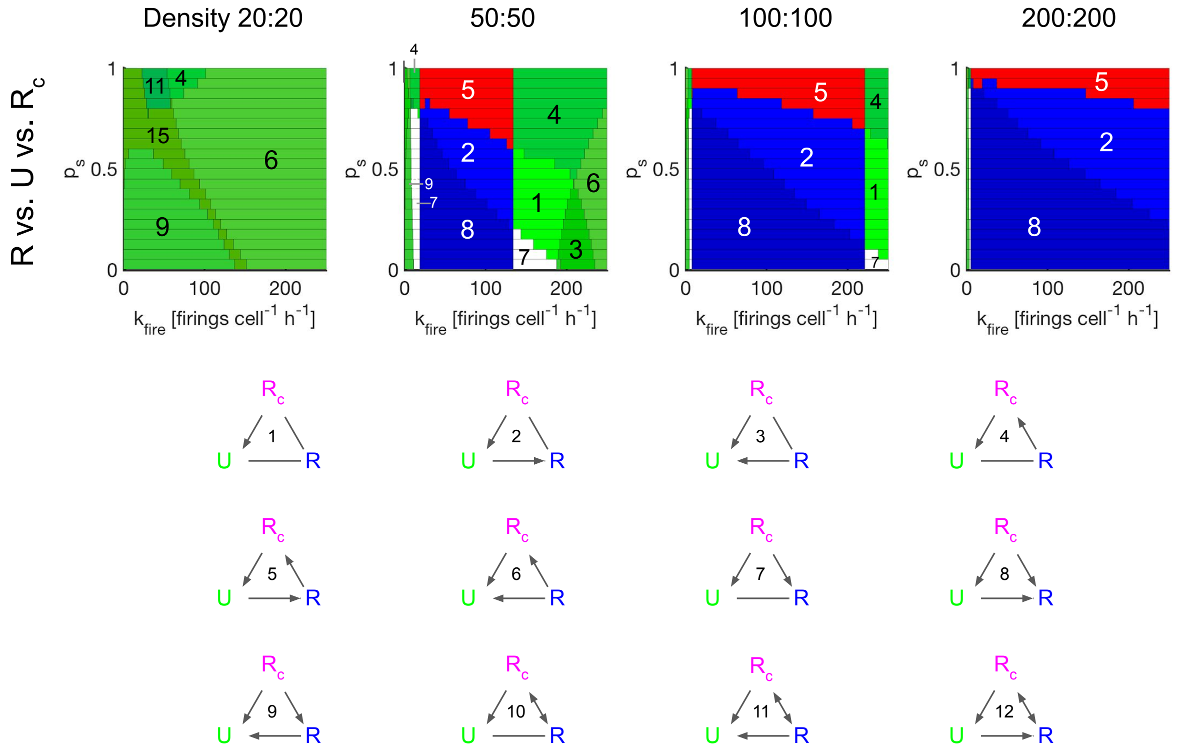

Supplementary Figure 9: Global invasion plots (U vs R), with variable T6SS firing rate k_{fire} and self-immunity probability p_s, in the presence of a 'cheater' strategist R_c. Columns in the grid correspond to different cell densities tested (initial cell counts 20:20, 50:50, 100:100 and 200:200). Each plot is color-coded, with colored zones numbered 1-12. The arrow diagrams below the plots depict the invasion behavior within each numbered zone. Arrows point towards strategist(s) that can invade competitors from rarity. Plots constructed using fitness values (mean of 5 replicates) for 8 k_{fire} values with 7 competitor pairings; 280 simulations per plot. Parameters: $k_{\text{lysis}} = 8.0$ h^{-1}, $c = 0.001$, $N_{\text{hits}} = 2$. Source data are provided as a Source Data file.

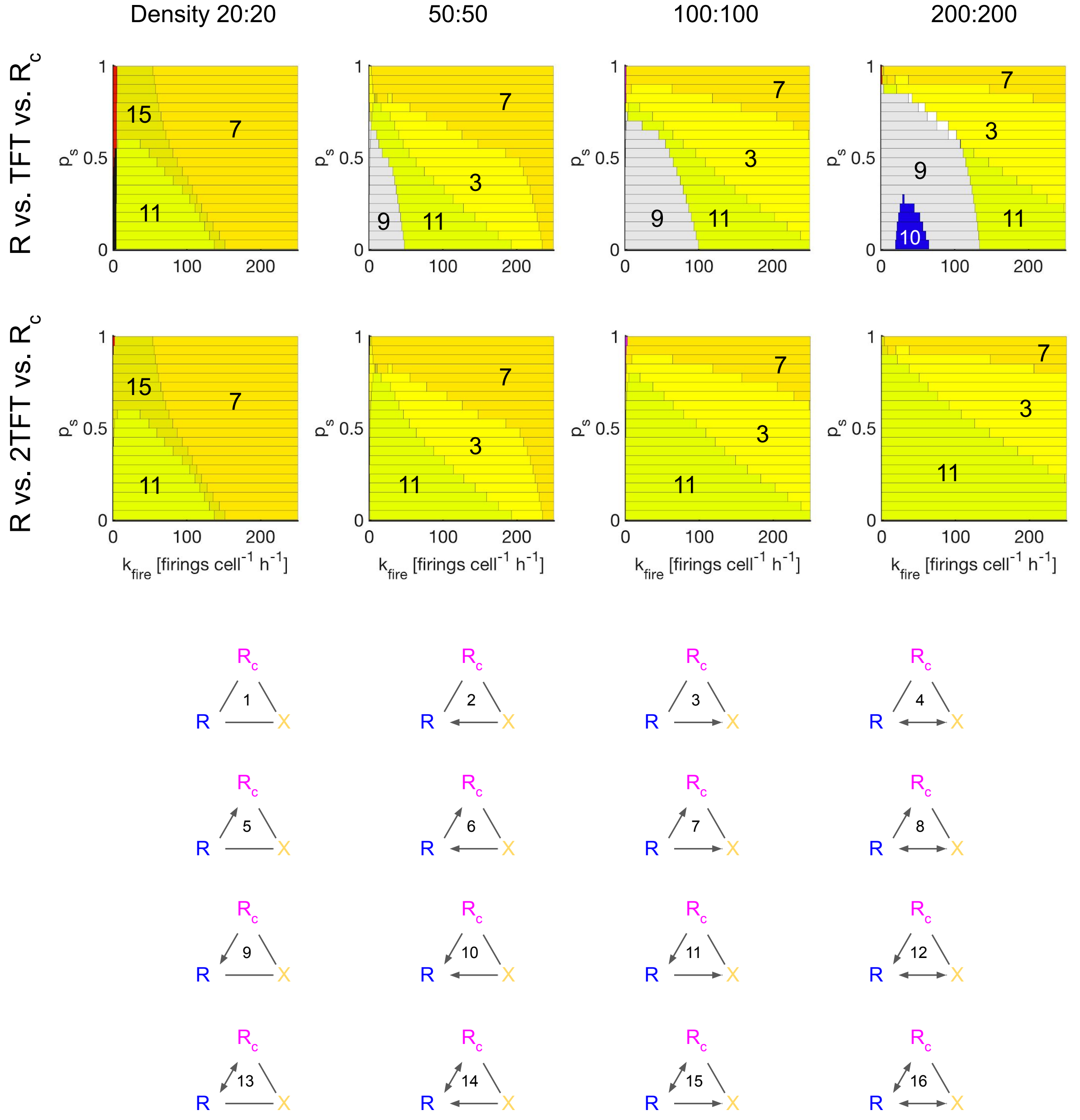

Supplementary Figure 10: Global invasion plots (R vs. TFT or 2TFT) for variable T6SS firing rate k_{fire} and self-immunity probability p_ss, in the presence of a 'cheater' strategist R_c. Columns in the grid correspond to different cell densities tested (initial cell counts 20:20, 50:50, 100:100 and 200:200); rows depict competitions between different triads of strategists. Each plot is color-coded, with colored zones numbered 1-16. The arrow diagrams below the plots depict the invasion behavior within each numbered zone. Arrows point towards strategist(s) that can invade competitors from rarity. Competitor 'X' corresponds to either TFT (top row) or 2TFT (bottom row). Plots constructed using fitness values (mean of 5 replicates) for 8 k_{fire} values with 9 competitor pairings; 360 simulations per plot. Parameters: k_{lysis} = 8.0 h^{-1}, c = 0.001, N_{hits} = 2. Source data are provided as a Source Data file.

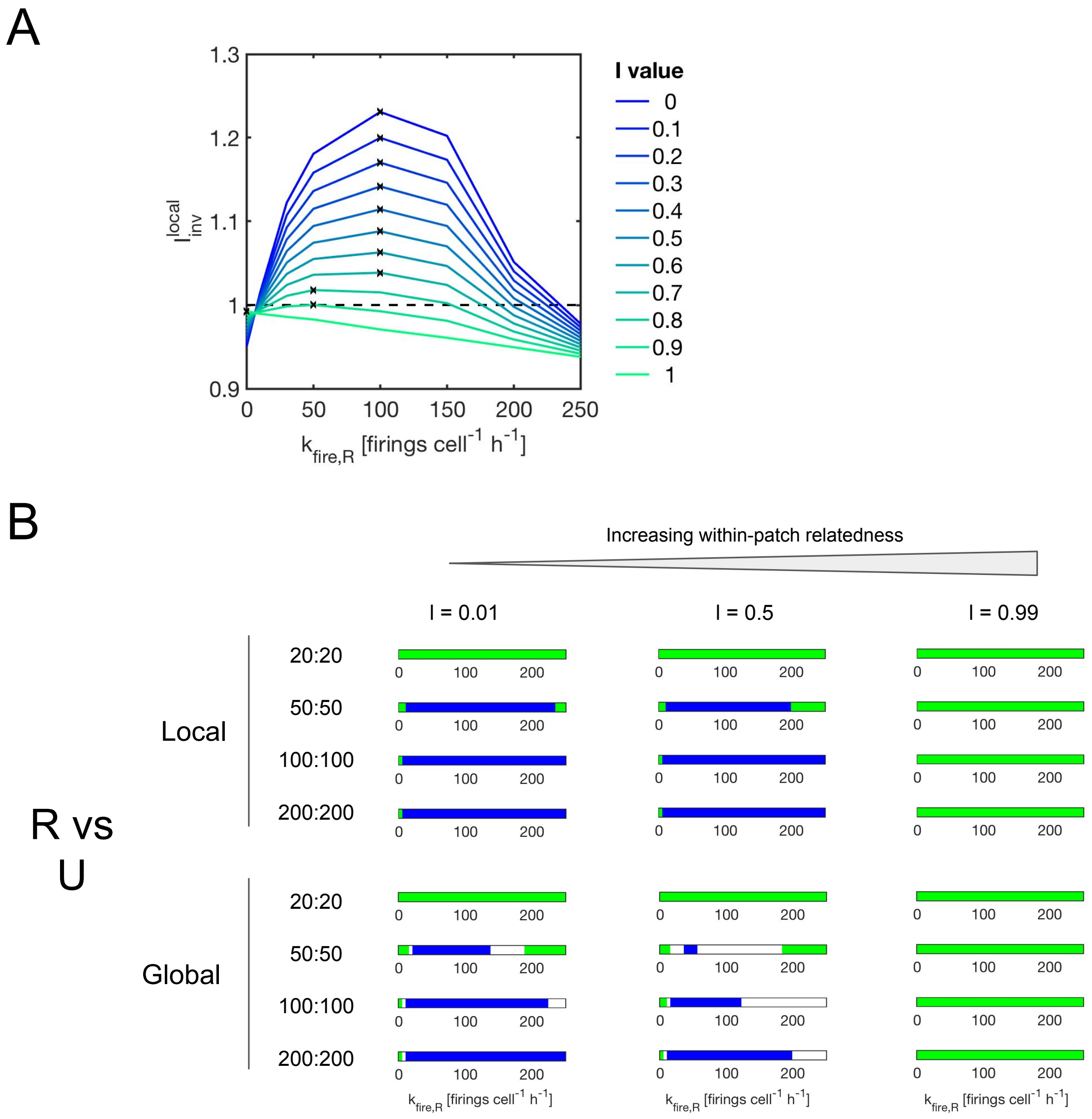

Supplementary Figure 11: Effects of increasing within-patch relatedness on R vs U competition. (**A**) Local invasion index ($I_{\text{inv}}^{\text{local}}$) traces plotted against R cell T6SS firing rate $k_{\text{fire,R}}$ for increasing values of within-patch relatedness (parameterized by isolation probability I; see Materials and Methods). For each trace, we mark the firing rate value that maximizes $I_{\text{inv}}^{\text{local}}$, to show that the optimum firing rate against an unarmed attacker generally falls with increasing within-patch relatedness. (B) 1-D local and global invasion plots for various initial cell densities, analogous to those shown in Supplementary Figure 3, recomputed for increasing values of I (columns). Parameters: $k_{\text{lysis}} = 8.0$ h^{-1}, $c = 0.001$, $N_{\text{hits}} = 2$. Source data are provided as a Source Data file.

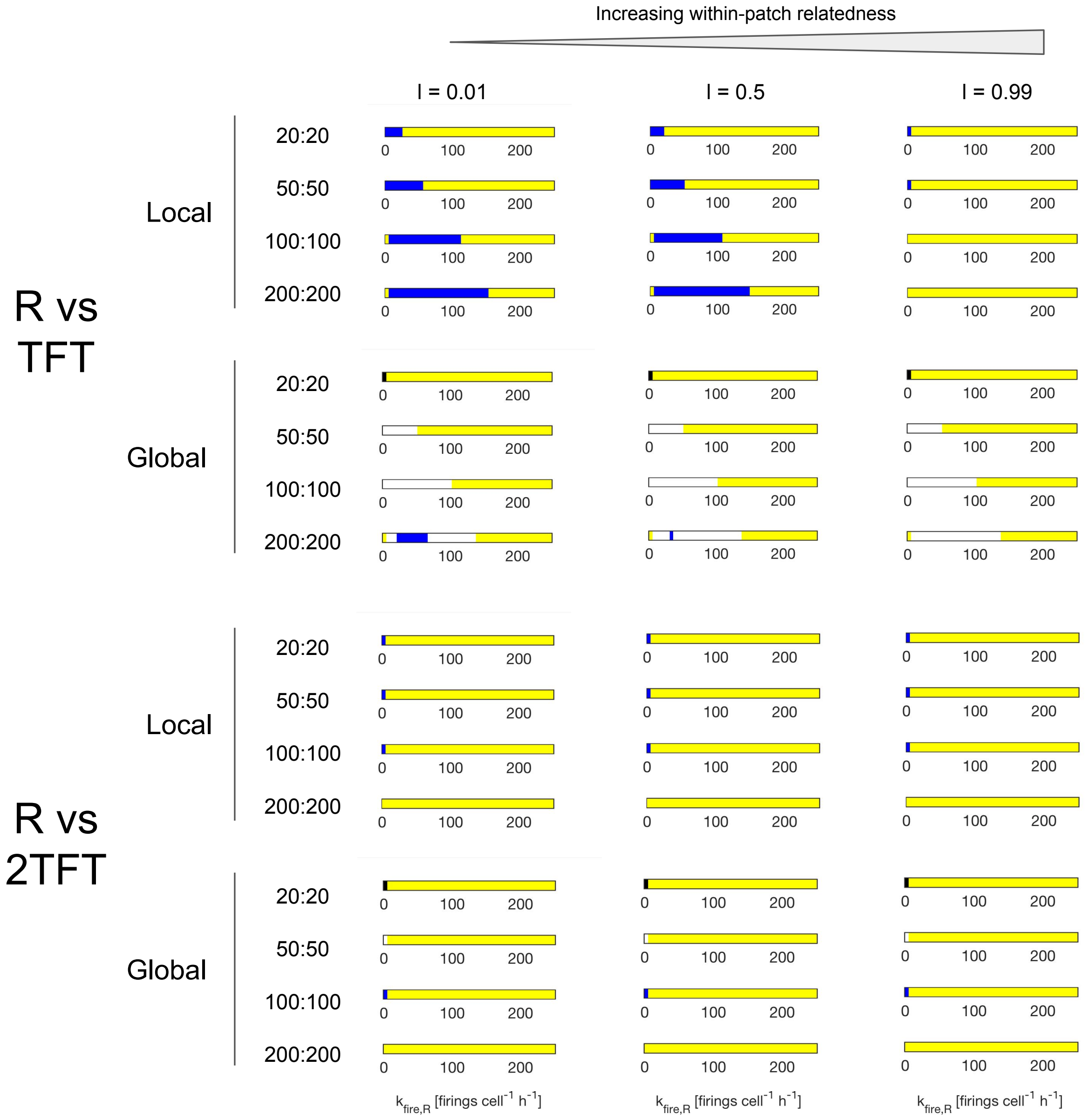

Supplementary Figure 12: Effects of increasing within-patch relatedness on R vs TFT, 2TFT competition. 1-D local and global invasion plots shown for various initial cell densities, analogous to those shown in Supplementary Figure 3, recomputed for increasing values of within-patch relatedness (parameterized by isolation probability I; see Materials and Methods). Parameters: $k_{\text{lysis}} = 8.0$ h^{-1}, $c = 0.001$, $N_{\text{hits}} = 2$. Source data are provided as a Source Data file.

Supplementary References

1. P. C. Kirchberger, D. Unterweger, D. Provenzano, S. Pukatzki, Y. Boucher, Sequential displacement of Type VI Secretion System effector genes leads to evolution of diverse immunity gene arrays in Vibrio cholerae. *Sci. Rep.* **7**, 45133 (2017).
2. K. D. LaCourse *et al.*, Conditional toxicity and synergy drive diversity among antibacterial effectors. *Nat. Microbiol.* **3**, 440–446 (2018).
3. T. J. Rudge, P. J. Steiner, A. Phillips, J. Haseloff, Computational Modeling of Synthetic Microbial Biofilms. *ACS Synth. Biol.* **1**, 345–352 (2012).
4. M. Basler, B. T. Ho, J. J. Mekalanos, Tit-for-Tat: Type VI Secretion System Counterattack during Bacterial Cell-Cell Interactions. *Cell.* **152**, 884–894 (2013).
5. W. P. J. Smith *et al.*, Cell morphology drives spatial patterning in microbial communities. *Proc. Natl. Acad. Sci.*, E280–E286 (2016).
