# Supplementary material for: The evolution of tit-for-tat in bacteria via the type VI secretion system
Source: Nat Commun. 2020 Oct 26;11:5395. doi: 10.1038/s41467-020-19017-z (PMC7589516; doi:10.1038/s41467-020-19017-z)
Supplement: Supplementary file 2 — Reporting Summary [file 41467_2020_19017_MOESM2_ESM.pdf]

nature research

Corresponding author(s): Kevin R. Foster

Last updated by author(s): Sep 16, 2020

Reporting Summary

Nature Research wishes to improve the reproducibility of the work that we publish. This form provides structure for consistency and transparency in reporting. For further information on Nature Research policies, see our Editorial Policies and the Editorial Policy Checklist.

Statistics

For all statistical analyses, confirm that the following items are present in the figure legend, table legend, main text, or Methods section.

n/a	Confirmed	
☐	☒	The exact sample size (n) for each experimental group/condition, given as a discrete number and unit of measurement
☐	☒	A statement on whether measurements were taken from distinct samples or whether the same sample was measured repeatedly
☐	☒	The statistical test(s) used AND whether they are one- or two-sided<br>*Only common tests should be described solely by name; describe more complex techniques in the Methods section.*
☒	☐	A description of all covariates tested
☐	☒	A description of any assumptions or corrections, such as tests of normality and adjustment for multiple comparisons
☐	☒	A full description of the statistical parameters including central tendency (e.g. means) or other basic estimates (e.g. regression coefficient) AND variation (e.g. standard deviation) or associated estimates of uncertainty (e.g. confidence intervals)
☐	☒	For null hypothesis testing, the test statistic (e.g. F, t, r) with confidence intervals, effect sizes, degrees of freedom and P value noted<br>*Give P values as exact values whenever suitable.*
☒	☐	For Bayesian analysis, information on the choice of priors and Markov chain Monte Carlo settings
☒	☐	For hierarchical and complex designs, identification of the appropriate level for tests and full reporting of outcomes
☐	☒	Estimates of effect sizes (e.g. Cohen's d, Pearson's r), indicating how they were calculated

Our web collection on statistics for biologists contains articles on many of the points above.

Software and code

Policy information about availability of computer code

Data collection	Simulation data were collected using the open source modeling tool CellModeller (V4.0) with a custom module added to simulate T6SS activity (code available for download at https://github.com/WilliamPJSmith/CellModeller). Bacterial cells were imaged on an inverted Nikon Ti Eclipse epifluorescence microscope and recorded on a pco.edge 4.2 sCMOS camera using commercial VisiView software (V4.4.0.10).
Data analysis	Simulation data were processed using MATLAB (R2017b) and visualized using Paraview open source vizualisation software (V5.4.0). Recorded time-lapse series were post-processed using Fiji (V1.0); statistical tests were performed with GraphPad Prism (V7.0) and MATLAB (Version R2017a 9.2.0.556344).

For manuscripts utilizing custom algorithms or software that are central to the research but not yet described in published literature, software must be made available to editors and reviewers. We strongly encourage code deposition in a community repository (e.g. GitHub). See the Nature Research guidelines for submitting code & software for further information.

Data

Policy information about availability of data

All manuscripts must include a data availability statement. This statement should provide the following information, where applicable:

- Accession codes, unique identifiers, or web links for publicly available datasets
- A list of figures that have associated raw data
- A description of any restrictions on data availability

Source data are provided with this paper.

Field-specific reporting

Please select the one below that is the best fit for your research. If you are not sure, read the appropriate sections before making your selection.

- [x] Life sciences
- [ ] Behavioural & social sciences
- [ ] Ecological, evolutionary & environmental sciences

For a reference copy of the document with all sections, see nature.com/documents/nr-reporting-summary-flat.pdf

Life sciences study design

All studies must disclose on these points even when the disclosure is negative.

Sample size	No sample size calculation was carried out prior to data collection. Based on work published previously (see Smith, William PJ, et al. "The evolution of the type VI secretion system as a disintegration weapon." Plos Biology 18.5 (2020): e3000720), default starting samples of 5 and 2 replicates were used for simulations and experiments, respectively. Following simulation data collection, sample means were tested using a convergence criterion, verifying negligible change in means following random downsampling (see Smith, William PJ, et al. "Cell morphology drives spatial patterning in microbial communities." PNAS 114.3 (2017): E280-E286). Sample sizes were increased until convergence was verified.
Data exclusions	During confocal analysis of retaliatory T6SS firing (P. aeruginosa + V. cholerae co-culture), we used the following pre-established exclusion criterion: T6SS structures within Pa cells were ignored where the Pa cell was not in contact with at least one Vc cell. This excluded Pa T6SS structures that could not have been formed in retaliation to an incident Vc attack.
Replication	All experimental data are representative of two independent biological replicates, carried out on different occasions. All replication attempts were successful.
Randomization	This experiment is not amenable to randomization. The two treatment groups are, by nature, visually distinguishable, rendering any treatment randomization or blinding pointless.
Blinding	This experiment is not amenable to blinding. The two treatment groups are, by nature, visually distinguishable, rendering any treatment randomization or blinding pointless. Moreover, the author who performed the experiments also analyzed the data.

Reporting for specific materials, systems and methods

We require information from authors about some types of materials, experimental systems and methods used in many studies. Here, indicate whether each material, system or method listed is relevant to your study. If you are not sure if a list item applies to your research, read the appropriate section before selecting a response.

Materials & experimental systems

n/a	Involved in the study	
x		Antibodies
x		Eukaryotic cell lines
x		Palaeontology and archaeology
x		Animals and other organisms
x		Human research participants
x		Clinical data
x		Dual use research of concern

Methods

n/a	Involved in the study	
x		ChIP-seq
x		Flow cytometry
x		MRI-based neuroimaging
