# Supplementary material for: The evolution of tit-for-tat in bacteria via the type VI secretion system
Source: Nat Commun. 2020 Oct 26;11:5395. doi: 10.1038/s41467-020-19017-z (PMC7589516; doi:10.1038/s41467-020-19017-z)
Supplement: Supplementary file 3 — Description of Additional Supplementary Files [file 41467_2020_19017_MOESM3_ESM.pdf]

Description of Additional Supplementary Files

Supplementary Movie 1: Simulated competitions between two T6SS- (U)narmed strains. Three representative agent-based model simulations show monolayer 'patch' communities growing from a randomly-scattered 1:1 inoculum of two unarmed strains (green, dark green). From left to right, the inoculation cell density is increased (20:20, 50:50 and 100:100 cells scattered in a circle of diameter 100.0 µm), resulting in greater contact between the two strains. Each patch contains a fixed quota of growth-limiting resources, which cells consume as they divide exponentially. Simulations terminate once patch resources are depleted. Movie plays at 15 frames per second; screen measures ~750 x 450 µm.

Supplementary Movie 2: Simulated competitions between T6SS- (U)narmed and T6SS+ (R)andom-firing strains. Three representative agent-based model simulations show competition between an unarmed strain (green) competing with a T6SS+ random-firing strain (blue); intoxicated cells are shown in red. Increasing initial cell density (left to right: 20:20, 50:50. 100:100 cells) increases U-R strain contact, resulting in increased killing of U-type cells. Parameters used throughout: N_{hits}= 2, $k_{\mathrm{fire,R}}$ = 50.0 firings cell^{-1} h^{-1}, k_{lysis} = 8.0 h^{-1}, c_{upfront} = 0.05, c = 0.001. Movie plays at 15 frames per second; screen measures ~750 x 450 µm.

Supplementary Movie 3: Simulated competitions between two T6SS+ (R)andom-firing strains. Three representative agent-based model simulations show competition between two T6SS+ random-firing strains (R1: pink; R2, blue) at fixed cell density (200:200 cell inoculum).

The two strains are mutually susceptible to one another's toxins. From left to right, R1's firing rate is held constant ($k_{\mathrm{fire,R1}}$ = 50.0 firings cell^{-1} h^{-1},) while R2's firing rate is increased ($k_{\mathrm{fire,R2}} = 0.0$, 50.0 and 200.0 firings cell^{-1} h^{-1}). N_{hits}= 2, k_{lysis} = 8.0 h^{-1}, c_{upfront} = 0.05, c = 0.001. Movie plays at 15 frames per second; screen measures ~750 x 450 µm.

Supplementary Movie 4: Simulated competitions between T6SS+ (R)andom-firing and T6SS+ Tit-For-Tat (TFT) retaliator strains. Three representative agent-based model simulations show competition between a T6SS+ random-firing strain (blue) competing with a T6SS+ tit-for-tat retaliator strain (yellow); intoxicated cells are shown in red. The two strains are mutually susceptible to one another's toxins. Increasing initial cell density (left to right: 20:20, 50:50. 100:100 cells) increases inter-strain contact, favoring the random attacker. Parameters used throughout: N_{hits}= 2, $k_{\mathrm{fire,R}}$ = 50.0 firings cell^{-1} h^{-1}, k_{lysis} = 8.0 h^{-1}, c_{upfront} = 0.05, c = 0.001. Movie plays at 15 frames per second; screen measures ~750 x 450 µm.

Supplementary Movie 5: Simulated competitions between T6SS+ (R)andom-firing and T6SS+ 2-Tits-For-Tat (2TFT) retaliator strains. Three representative agent-based model simulations show competition between a T6SS+ random-firing strain (blue) competing with a T6SS+ 2-tits-for-tat retaliator strain (yellow); intoxicated cells are shown in red. The two strains are mutually susceptible to one another's toxins. Increasing initial cell density (left to right: 20:20, 50:50. 100:100 cells) increases inter-strain contact, favoring the retaliator strain. Parameters used throughout: N_{hits}= 2, $k_{\mathrm{fire,R}}$ = 50.0 firings cell^{-1} h^{-1}, k_{lysis} = 8.0 h^{-1}, c_{upfront} = 0.05, c = 0.001. Movie plays at 15 frames per second; screen measures ~750 x 450 µm.

Supplementary Movie 6: Overview of mixtures containing *P. aeruginosa* PAO1 and T6SS+ *V. cholerae* 2740-80. *P. aeruginosa* PAO1 *tssB-mNeongreen* was mixed with *V. cholerae* 2740-80 *vipA-mCherry2* in 1:5 ratio and monitored over 5 min at a frame rate of 30 frames per minute. Two representative time-lapse series are shown. A merge of phase contrast, GFP channel and mCherry channel is shown. Contrast was adjusted equally. Fields of view measure 39 x 26 µm; scale bar represents 5 µm. Movie plays at 5 frames per second.

Supplementary Movie 7: Overview of mixtures containing *P. aeruginosa* PAO1 and T6SS- *V. cholerae* 2740-80. *P. aeruginosa* PAO1 *tssB-mNeongreen* was mixed with *V. cholerae* 2740-80 *vipA-mCherry2 Δhcp1 Δhcp2* in 1:5 ratio and monitored over 5 min at a frame rate of 30 frames per minute. Two representative time-lapse series are shown. A merge of phase contrast, GFP channel and mCherry channel is shown. Contrast was adjusted equally. Fields of view measure 39 x 26 µm; scale bar represents 5 µm. Movie plays at 5 frames per second.

Supplementary Movie 8: Close-ups of mixtures containing *P. aeruginosa* PAO1 and T6SS+ *V. cholerae* 2740-80. *P. aeruginosa* PAO1 *tssB-mNeongreen* was mixed with *V. cholerae* 2740-80 *vipA-mCherry2* in 1:5 ratio and monitored over 5 min at a frame rate of 30 frames per minute. Examples showing between 1 and 6 *P. aeruginosa* T6SS firings against *V. cholerae* are presented (2 examples each). A merge of phase contrast, GFP channel and mCherry channel is shown. Contrast was adjusted equally. Fields of view measure 6.5 x 6.5 µm; scale bar represents 1 µm. Movie plays at 5 frames per second.
